# Chemotherapy-Induced Hematological Toxicity in Patients with Renal or Hepatic Impairment

**DOI:** 10.3390/pharmaceutics17101280

**Published:** 2025-09-30

**Authors:** Kelly Nies, Robin Vernooij, Lot Devriese, Jan-Hendrik Venhuizen, Maarten ten Berg, Christina Swart, Laureen Lammers, Saskia Haitjema

**Affiliations:** 1 Central Diagnostic Laboratory, University Medical Center Utrecht, Utrecht University, 3508 GA Utrecht, The Netherlands; k.p.h.nies-2@umcutrecht.nl (K.N.); r.w.m.vernooij-2@umcutrecht.nl (R.V.); j.h.s.venhuizen@umcutrecht.nl (J.-H.V.);; 2 Department of Nephrology and Hypertension, University Medical Center Utrecht, Utrecht University, 3508 GA Utrecht, The Netherlands; 3 Julius Center for Health Sciences and Primary Care, University Medical Center Utrecht, Utrecht University, 3508 GA Utrecht, The Netherlands; 4 Department of Medical Oncology, University Medical Center Utrecht, Utrecht University, 3508 GA Utrecht, The Netherlands; 5 Department of Clinical Pharmacy, University Medical Center Utrecht, Utrecht University, 3508 GA Utrecht, The Netherlands

**Keywords:** chemotherapy, renal impairment, hepatic impairment, hematological toxicity

## Abstract

**Background/Objectives**: Hematological toxicities (i.e., neutropenia, thrombocytopenia, and anemia), are common chemotherapy complications and may be exacerbated by renal or hepatic impairment due to altered drug exposure. This study assessed the association between renal and hepatic impairment and hematologic toxicities during chemotherapy in routine clinical practice. **Methods**: A single-center retrospective cohort study using the Utrecht Patient Oriented Database (UPOD) identified all chemotherapy administrations at the University Medical Centre Utrecht between 2011 and 2024. Regimens administered in ≥10 patients and ≥5 renally (GFR < 60 mL/min) or hepatically (bilirubin or AST > 1× ULN) impaired patients were included in descriptive analyses. Cox proportional hazards models estimated associations between organ impairment and grade ≥ 3 hematologic toxicities for regimens with ≥10 events per toxicity endpoint. **Results**: Overall, 4489 patients were included in renal analyses and 6218 in hepatic analyses, with smaller endpoint-specific subgroups for survival analyses. Renal impairment was associated with grade ≥ 3 neutropenia (HR: 1.43 [95% CI: 1.18–1.73]), thrombocytopenia (HR: 1.46 [95% CI: 1.15–1.86], and anemia (HR: 1.66 [1.27–2.16]). Hepatic impairment was similarly associated with neutropenia (HR: 1.25 [95% CI: 1.11–1.40]), thrombocytopenia (HR: 1.33 [95% CI: 1.13–1.57]), and anemia (HR: 1.62 [95% CI: 1.34–1.95]). Cyclophosphamide (pro-drug) regimens showed higher toxicity risk in renally impaired patients and reduced risk in hepatically impaired patients. Etoposide, melphalan and methotrexate were associated with increased toxicity in hepatically impaired patients. **Conclusions**: Renal and hepatic impairment significantly increase chemotherapy-induced hematologic toxicity. Several high-risk chemotherapy regimens were identified; however, larger multi-center datasets are needed to refine dosing guidance based on renal and hepatic function.

## 1. Introduction

Chemotherapeutic agents are important compounds in the treatment of many solid and hematological malignancies. However, treatment efficacy is dependent on narrow therapeutic windows and affected by dose-limiting toxicities. Careful determination of the maximum tolerated dose is essential to balance treatment efficacy and safety, minimizing the risk of life-threatening adverse effects. One of the most common and clinically significant adverse effects is severe hematological toxicity or myelosuppression, which can manifest as neutropenia, thrombocytopenia, or anemia. The incidence and severity of these toxicities varies significantly based on tumor type, treatment regimen, and patient comorbidities, for instance, in metastatic colorectal cancer, severe (grade ≥ 3) neutropenia, anemia, and thrombocytopenia occurred in 35.1%, 20.4%, and 8.1%, respectively [1]. In a broader retrospective study including various therapy regimen and clinical indications, moderate to severe (grade ≥ 2) hematologic toxicity was observed in 53.9% of patients, including neutropenia in 45.1% and anemia in 22.4% of patients [2]. These toxicities carry substantial risks with increased susceptibility to infections and sepsis for neutropenia [3], severe thrombocytopenia predisposes patients to bleeding complications including intracranial hemorrhage [4], and severe anemia contributes to fatigue and cardiac strain [5]. Hematological toxicity is a leading cause of chemotherapy dose reductions, delays, or treatment discontinuation, which may compromise treatment efficacy and negatively impact progression-free and overall survival [6].

The maximum tolerated dose of a chemotherapeutic agent is established in clinical trials within a controlled, homogeneous population. Patients with significant comorbidities or organ dysfunction, including renal or hepatic impairment, or multiple lines of previous chemotherapy, are systematically excluded from such trials [7]. As a result, standard dosing recommendations are frequently extrapolated from populations in trials that do not represent the medically complex real-world cancer population. This mismatch can be especially detrimental for patients with renal or hepatic impairment, who may experience different drug handling and toxicity-efficacy balances than those anticipated by trial-based guidelines. For instance, renal impairment, characterized by reduced glomerular filtration and tubular secretion, can prolong drug retention and increase systemic exposure for renally excreted agents such as cisplatin and methotrexate [8]. Hepatic impairment, in turn, can lead to slower metabolism of drugs processed by cytochrome P450 (CYP450) including taxanes and anthracyclines, resulting in increased plasma concentrations and prolonged half-life [9]. On the other hand, hepatic impairment may impede prodrug activation, potentially reducing treatment efficacy. The Renal Insufficiency and Anticancer Medications (IRMA) study reported that 50–60% of patients with solid tumors had renal insufficiency, driven by aging demographics, disease-related organ dysfunction, or prior treatment toxicity [10]. The prevalence of pre-existing hepatic impairment in cancer patients has not been reported in a large-scale observational study to our knowledge. Therefore, robust data on the impact of organ impairment on chemotherapy outcomes is crucial for enhancing patient safety and treatment efficacy.

Since renal and hepatic function have a large impact on drug exposure and pharmacokinetics, dose adjustment in patients with impaired renal or hepatic function may be needed to assure the treatment will stay within an optimal therapeutic window while maintaining patient safety. Current recommendations on dose adjustments for these populations are mainly based on a small number of patients, limited clinical trial data, or in some cases solely on theoretical pharmacokinetic expectations regarding renal and hepatic clearance pathways of the particular chemotherapeutic agent [6]. Real-world evidence remains sparse, particularly for combination regimens commonly used in routine care. Therefore, there is an urgent need to evaluate hematological toxicity patterns in these vulnerable subpopulations to facilitate optimized chemotherapeutic regimen selection and dose modification. In this study, we analyze routine care data to assess the association between renal and hepatic function and hematological toxicity across diverse chemotherapeutic protocols, aiming to inform evidence-based dose individualization.

## 2. Materials and Methods

### 2.1. Study Setting

This retrospective, single-center study was conducted at the University Medical Centre Utrecht (UMC Utrecht), a tertiary referral hospital located in the Netherlands. Data were extracted from the Utrecht Patient Oriented Database (UPOD), which contains prospectively collected data including patient information, medication orders, and laboratory test results [11]. Chemotherapy regimen details on administrated compounds, dosage, and administration frequency were retrieved from BD Cato™, the hospital’s oncology prescription software. Regimens including at least one parenteral cytotoxic agent were prescribed via BD Cato™.

### 2.2. Study Population

Adult patients (≥18 years) who received cytostatic agents prescribed via BD Cato™ at the UMCU between January 2011 and December 2024 were eligible for inclusion. To avoid interference from prior treatment with cytostatic agents, only the first treatment course per patient was evaluated. Patients were excluded if (1) no hematological laboratory values were available within ≤14 days prior to the start of the chemotherapy protocol, (2) grade 3–4 hematological abnormalities were present at baseline, which was defined as leukopenia (<2.0 × 10^9^/L), neutropenia (<1.0 × 10^9^/L), thrombocytopenia (grade 3–4: <50 × 10^9^/L) and/or anemia (grade 3–4: <4.9 mmol/L) in accordance with the Common Terminology Criteria for Adverse Events (CTCAE) version 5.0 [12], or (3) the initial chemotherapy dose deviated ≥15% from the standard protocol, reflecting an upfront dose-adjustment, since such deviations may reflect prior treatment in a different hospital, reduced overall survival, and skewed toxicity risk.

This study was not subject to the Medical Research Involving Human Subjects Act (WMO) as determined by the Medical Research Ethics Committee of the UMCU. Ethical approval or informed consent was waived as obtaining this would be a disproportionate effort because of high patient numbers. European privacy law and local guidelines were respected.

### 2.3. Outcome and Parameter Definitions

Renal functioning was estimated based on glomerular filtration rate (eGFR) as determined prior to the start of the treatment regimens conform routine clinical practice. Consequently, two equations were used, i.e., the MDRD (January 2011–September 2013) and the CKD-EPI 2009 (October 2013–December 2024). Patients were categorized into mild (45–59 mL/min GFR), moderate (30–44 mL/min GFR), and severe (<30 mL/min GFR) renal impairment. Renal impairment was defined as mild to severe impairment based on eGFR < 60 mL/min/1.73 m^2^.

While the Child-Pugh classification is commonly used for defining hepatic impairment, its components including serum albumin, ascites, and hepatic encephalopathy, can be affected by cancer-related conditions like metastatic disease or cachexia [13]. For this reason, the National Cancer Institute (NCI) criteria, based on total bilirubin and aspartate aminotransferase (AST) levels, are more appropriate in oncology and were therefore used in this study [14]. Mild hepatic impairment was defined as a bilirubin > 1.0x ULN and/or AST > 1.0x ULN, which translated to total bilirubin > 21 mmol/L and AST > 30 mmol/L for females and AST > 35 mmol/L for males. Moderate and severe hepatic impairment were defined as total bilirubin 1.5–3x ULN and >3 ULN, respectively.

The primary outcome included grade 3–4 hematologic toxicity defined as leukopenia (<2.0 × 10^9^/L), neutropenia (<1.0 × 10^9^/L), thrombocytopenia (grade 3–4: <50 × 10^9^/L) and/or anemia (grade 3–4: <4.9 mmol/L) in accordance with CTCAE version 5.0 [12].

Primary tumor site was identified primarily from tumor diagnoses according to the International Classification of Diseases, Tenth Revision (ICD-10) or, alternatively, diagnosis treatment combinations (the Dutch case-mix system) registered for health care insurers was used. If neither was available, the tumor site was determined based on the cancer type specified in the chemotherapy regimen dosing schedule.

### 2.4. Statistical Analyses

Baseline patient characteristics were reported as median (interquartile range) for continuous variables, as the Shapiro–Wilk test indicated all continuous variables were non-normally distributed. Mann–Whitney U-tests were applied to compare continuous variables. Pearson’s chi-square or, alternatively, Fisher’s exact test (if expected counts < 5) were used for categorical variables.

Time-to-event analyses were conducted to assess the association between organ impairment and hematologic toxicity. The following variables were considered potential confounders: age, sex, and chemotherapy regimen. Age and sex were selected because they can influence pharmacokinetics and susceptibility to toxicity [15,16,17]. Chemotherapeutic regimens were defined as the combination of administered cytotoxic compound(s), which could consist of a single cytotoxic agent (monotherapy) or multiple agents (combination therapy). Regimens with the same combination of cytotoxic compounds were grouped together, irrespective of differences in administration frequency or dosage. Chemotherapy regimen was selected as a potential confounder because of compound-specific differences in hematological toxicity risk [18,19]. Cox proportional hazards models were applied unadjusted (model 1), adjusted for age and sex (model 2), and adjusted for the administered chemotherapy compound or compound combination (model 3). Chemotherapy compounds or compound combinations administered in <10 patients, with <10 toxicity events (neutropenia, thrombocytopenia, or anemia), or with <5 patients with an impaired renal or hepatic function were excluded to reduce risk of overfitting. This resulted in differently sized datasets for survival analysis of each hematologic toxicity endpoint. Additionally, separate survival analyses were performed per chemotherapeutic compound or compound combination to estimate hazard ratios of impaired renal or hepatic function on the incidence of hematologic toxicity.

#### Sensitivity Analyses

A sensitivity analysis excluded patients treated with a chemotherapy compound for which renal or hepatic clearance is not expected to significantly influence drug exposure or toxicity. Compound selection has been established under the supervision of an experienced clinical pharmacologist and was based on previously reported pharmacokinetic literature and recommendations on dose modifications for renal and hepatic impairment [6] (Supplementary A, Tables S1–S2). Where uncertainty existed regarding metabolism and clearance, the compound was retained in the analyses to avoid unjustified exclusions. Cox proportional hazards models adjusted for chemotherapy regimen (model 3) were repeated in a restricted cohort of patients receiving any of the chemotherapy compounds with known or uncertain renal/hepatic elimination pathways (model 4).

## 3. Results

**Figure 1:**
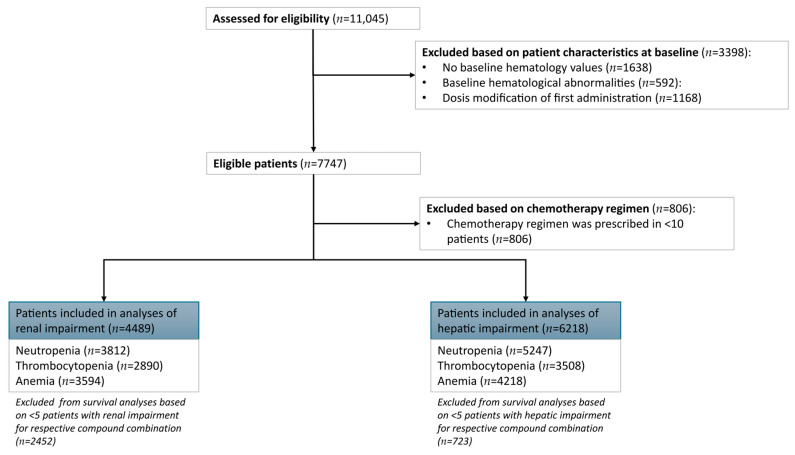
Flowchart of included patients. Only patients treated with a chemotherapy protocol administered in ≥5 patients with renal or hepatic impairment were included in their respective descriptive analyses. For survival analyses per outcome of interest (grade ≥ 3 neutropenia, thrombocytopenia, and anemia) a minimum of 10 events per chemotherapy protocol was needed, therefore leading to different subsets for survival analyses of each of the hematologic toxicities.

In total 11,145 patients received chemotherapy during the inclusion period (Figure 1). The absence of baseline measurements of hematological laboratory values led to the exclusion of 1638 patients. Another 592 patients had hematological abnormalities at baseline and were excluded from further analysis. Dose modifications at baseline led to the exclusion of 1168 patients. Out of 7747 patients with eligible baseline criteria, 806 patients received a chemotherapy compound combination that was administered in <10 patients, leading to exclusion.

Analyses were limited to chemotherapy compound combinations with at least five patients with renal or hepatic impairment, leading to the exclusion of 2452 patients from the renal impairment survival analyses and 723 patients for the hepatic impairment survival analyses. Therefore, the total dataset available for descriptive analytics of normal renal function versus renal impairment was 4489 patients and for normal hepatic function versus hepatic impairment was 6218 patients. After enforcing a minimum of 10 events per regimen, the renal impairment survival analysis datasets included 3812 for neutropenia as endpoint, 2890 for thrombocytopenia, and 3594 for anemia. For hepatic impairment survival analyses, data from 5247 patients was available to evaluate the neutropenia endpoint, 3508 for thrombocytopenia, and 4218 for anemia. Descriptive statistics for each analytic subset are provided in Supplementary A (Tables S3–S8).

The dataset suitable for the analysis of renal impairment in relation to hematologic toxicity consisted of in total 4489 patients with an equal distribution of male versus female patients and a median age of 62 (IQR: 53–69) years old (Table 1). Patients with a renal impairment were more often male (56% versus 51%) and of higher age with a difference of 7 years in the median age when compared to patients without renal impairment. Laboratory values of ALT and albumin were slightly lower for patients with renal impairment. Regarding baseline hematological values, significant differences in leukocyte and neutrophil count, and Hb were determined. Significant differences in the incidence of grade ≥ 3 hematological toxicity during the treatment regimen were determined for patients with renal impairment versus patients with no renal impairment. In total 22 different cytotoxic compound combinations are included in the renal analyses, with the top 5 most frequently administered being cisplatin (*n* = 990, 22%), carboplatin + paclitaxel (*n* = 706, 16%), pembrolizumab (*n* = 394, 8.8%), ipilimumab + nivolumab (*n* = 262, 5.8%), cyclophosphamide (*n* = 198, 4.4%), cyclophosphamide + doxorubicin + rituximab + vincristine (*n* = 182, 4.1%). An extended table (Supplementary A, Table S9) detailing all chemotherapy regimens included in the renal impairment analyses is provided.

Exclusion of chemotherapy regimens with <5 patients with an impaired hepatic function, resulted in exclusion of 742 patients, leading to a definitive dataset of 6199 patients. Almost all patients with hepatic impairment were categorized at mild hepatic impairment (*n* = 1182), while patients with moderate (*n* = 44) to severe (*n* = 15) hepatic impairment were sparsely represented. Therefore, descriptives of patients from all severities of hepatic impairment were grouped and compared to patients with no hepatic impairment. This dataset, suitable for the analyses of impaired hepatic function in relation to hematological toxicity, had a similar distribution of age comparing patients with and without hepatic impairment (Table 2). Also, for this dataset, significant differences were observed in all baseline laboratory and hematological measurements, except for the thrombocyte count. The incidence of hematological toxicity during the treatment regimen differed significantly between patients with hepatic impairment versus patients without hepatic impairment. Included in the hepatic impairment analyses were in total 55 different cytotoxic compound combinations, with some of the most frequent being cisplatin (*n* = 990, 16%), carboplatin + paclitaxel (*n* = 706, 11%), pembrolizumab (*n* = 394, 6.3%), ipilimumab + nivolumab (*n* = 262, 4.2%), bleomycin + cisplatin + etoposide (*n* = 236, 3.8%), cyclophosphamide + docetaxel + epirubicine + fluorouracil (*n* = 202, 3.2%). An extended table (Supplementary A, Table S10) detailing all chemotherapy regimens included in the hepatic impairment analyses is provided.

### 3.1. Risk of Hematological Toxicity in Patients with Mild Impairment of Renal Function

First, the association of dichotomized renal function was analyzed in relation to the different outcomes of interest (grade ≥ 3 neutropenia, thrombocytopenia, and anemia) (Table 3). Model 1, 2, and 3 included all chemotherapy regimens without prioritizing regimens that included compounds more likely to affect hematological toxicity due to renal clearance. By means of unadjusted Cox proportional Hazards models, mild to severe renal impairment (<60 mL/min eGFR) was found associated with an increased risk of neutropenia (HR: 1.43 [95% CI: 1.18–1.73]), thrombocytopenia (HR: 1.46 [95% CI: 1.15–1.86], and anemia (HR: 1.66 [1.27–2.16]). After adjusting for age and sex, the following HRs were found for neutropenia (HR: 1.54 [95% CI: 1.27–1.88]), thrombocytopenia (HR: 1.51 [95% CI: 1.19–1.92]), and anemia (HR: 1.86 [95% CI: 1.42–2.43]). When adjusting the model for the administered chemotherapy compound(s) (model 3), mild to severe renal impairment remained significantly associated with anemia (HR: 1.51 [1.15–1.99]).

Repeating the analysis in a subset of patients (*n* = 2929, Supplementary A, Table S11) who were treated with a preselected compound, resulted in higher HRs for all toxicities compared to the adjusted analyses performed in the total dataset (Table 3, model 4). Mild to severely impaired renal function in this subset of patients was found associated with neutropenia (HR: 1.44 [95% CI: 1.13–1.84]) and anemia (HR: 1.75 [1.24–2.46]).

To explore whether the severity of renal impairment differentially affected hematologic toxicity risk, patients were stratified into mild (GFR: 45–59 mL/min), moderate (GFR: 30–44 mL/min), and severe (GFR: <30 mL/min) renal impairment. The majority of patients (*n* = 4059) had no renal impairment (GFR: ≥60 mL/min), while mild, moderate, and severe impairment occurred in 269 (6%), 110 (2.5%), and 51 (1.1%) patients, respectively. In general, moderate renal impairment was associated with higher hazard ratios (HRs) for all hematologic toxicities compared to mild impairment. Severe renal impairment was only significantly associated with anemia, likely due to a relatively small sample size. Full results are provided in Supplementary A (Table S12).

### 3.2. Individual Chemotherapy Compounds and the Effect of Renal Function on Hematotoxicity

The risk of hematological toxicity was also explored per administered compound or combination therapy to provide insight into specific high-risk protocols for patients with impaired renal function (Table 4). Individual compounds and combination therapies from the list of selected compounds that are suspected to exert an impact on hematotoxicity, especially in patients with renal impairment, were analyzed separately. The most frequently administered protocol was a cisplatin monotherapy protocol in 990 patients, however only 18 (1.8%) patients treated within this protocol had mild to severe renal impairment, resulting in <5 patients with renal impairment per outcome of interest. In patients treated with a combination therapy of cyclophosphamide (750 mg/m^2^) + doxorubicin (50 mg/m^2^) + rituximab (375 mg/m^2^) + vincristine (2 mg/m^2^) during 3–8 cycles, mild to severe renal impairment was associated with neutropenia (HR: 1.86 [95%: 1.11–3.11]) and anemia (HR: 2.53 [1.20–5.34]). This protocol was developed for the indication of mantle cell lymphoma and diffuse large B-cell lymphoma.

An even stronger association (HR: 5.57 [1.88–16.46]) between renal impairment and anemia was observed for the combination therapy consisting of cyclophosphamide + doxorubicin. Almost all patients (117/128, 91%) receiving the combination therapy of cyclophosphamide + doxorubicin, were treated for breast cancer with dosing schedules included 600 mg/m^2^ cyclophosphamide + 60 mg/m^2^ doxorubicin administered 1x a week for 2 to 3 weeks. Other analyses did not reach statistical significance. All descriptives per compound for the renal analyses are provided in Supplementary B.1. An extended overview with detailed hematologic toxicity event rates per chemotherapy compound, grouped by sex and organ impairment, is provided as Supplementary C.

### 3.3. Risk of Hematological Toxicity in Patients with Impaired Hepatic Function

For the unadjusted analyses, impaired hepatic function was found to be associated with chemotherapy-induced neutropenia (HR: 1.25 [95% CI: 1.11–1.40]), thrombocytopenia (HR: 1.33 [95% CI: 1.13–1.57]), and anemia (HR: 1.62 [95% CI: 1.34–1.95]) (Table 5). After adjusting for age and sex, similar HRs were reported for neutropenia (HR: 1.27 [95% CI: 1.13–1.43]), thrombocytopenia (HR: 1.38 [95% CI: 1.17–1.63]), and anemia (HR: 1.61 [95% CI: 1.34–1.94]. Upon adjusting for the administered chemotherapy compound(s), impaired hepatic function only remained associated with anemia (HR: 1.33 [95% CI: 1.10–1.61]).

Similar to the renal analysis, a short-list of compounds metabolized by the liver, and therefore likely associated with toxicity in patients with hepatic impairment, was created. An additional analysis was performed in the patient subset (*n* = 3462, Supplementary A, Table S13) treated with compounds from this short-list (Table 5, model 4). These analyses resulted in similar results compared to the full list of compounds with a HR of 1.34 [95% CI: 1.06–1.69] for developing anemia during the course of the chemotherapy treatment.

### 3.4. Individual Chemotherapy Compounds and the Effect of Hepatic Function on Hematotoxicity

The risk of hematotoxicity in patients with impaired hepatic function is expected to vary between chemotherapy regimens due to compound-specific differences in metabolisation (Table 6). A combination protocol of bleomycin + cisplatin + etoposide administered in 236 patients, resulted in an increased risk of thrombocytopenia (HR: 2.38 [1.08–5.20]) and anemia (HR: 3.46 [1.29–9.33]) in patients with impaired hepatic function versus normal hepatic function. Out of 76 patients treated with methotrexate, 44 developed neutropenia and 35 developed thrombocytopenia. Impaired hepatic function was associated with neutropenia (HR: 3.05 [1.57–5.91]) and thrombocytopenia (HR: 2.98 [1.39–6.41]) for patients treated with methotrexate. Similar to methotrexate, monotherapy with melphalan was associated with neutropenia (HR: 2.91 [1.42–5.98]) and thrombocytopenia (HR: 3.07 [1.49–6.31]). Monotherapy with doxorubicin was administered in 38 patients, of which 7 developed anemia during the treatment course. Impaired hepatic function was associated with anemia (HR: 15.06 [1.81–125.42]) for treatment with doxorubicin.

On the contrary, patients with impaired hepatic function who received the pro-drug cyclophosphamide monotherapy had a lower risk of developing neutropenia (HR: 0.30 [0.13–0.69]) and thrombocytopenia (HR: 0.32 [0.14–0.74]).

The incidence of patients having both renal and hepatic impairment was limited to 85 patients out of 6361 (1.3%) patients with an eligible chemotherapy regimen of >10 patients. An overview of hematological toxicity during the chemotherapy regimen split into renal and hepatic impairment, renal impairment only, hepatic impairment only, and no impairment has been made available in Supplementary A (Table S14).

## 4. Discussion

Patients with impaired renal or hepatic function are prone to prolonged and increased drug exposure and, consequently, hematological toxicity is expected to occur more frequently. In line with this expectation, our study confirmed the association of both renal and hepatic impairment to an increased risk of developing hematologic toxicity during chemotherapy. The findings of this study were compared to a review providing dose recommendations for several high-risk cytotoxic agents [6]. Current dose recommendations are generally limited to severe renal or hepatic impairment, while the current study identified significantly increased risks of hematological toxicity in a population of primarily mild to moderate organ impairment.

In the overall patient population included in this study, impaired renal function was consistently associated with an increased risk of grade ≥ 3 hematological toxicity. In the analyses adjusted for the chemotherapy regimen, mild to severely impaired renal function remained associated with neutropenia and anemia. Moderate renal impairment in general increased the risk of neutropenia, thrombocytopenia, and anemia in comparison to mild renal impairment. Severe renal impairment was subject to a small sample size, which may explain why only the risk of anemia was significantly higher in severe versus mild renal impairment. Patients with mild to severe renal impairment who were treated with a combination protocol of cyclophosphamide + doxorubicin had a >5-fold increased risk of developing grade 3–4 anemia. Most patients (>90%) receiving this treatment combination were treated with 600 mg/m^2^ cyclophosphamide and 60 mg/m^2^ doxorubicin for breast cancer. This is in line with a study performed in 95 breast cancer patients receiving the same compound combination, which observed an odds ratio of 5.29 (95% CI: 2.10–13.33) in patients with <75 mL/min/1.73 m^2^ for experiencing an episode of neutropenic fever with hospital admission, treatment delay, dose adjustment due to toxicity, or the need for granulocyte colony-stimulating factor (G-CSF) [20]. Similar to our study population, this effect was observed in a population consisting of primarily mild renal impairment. A more extensive regimen consisting of cyclophosphamide (750 mg/m^2^) + doxorubicin (50 mg/m^2^) + rituximab (375 mg/m^2^) + vincristine (2 mg/m^2^) for the treatment of mantle cell lymphoma and diffuse large B-cell lymphoma resulted in a >2.5-fold increase in risk of anemia and close to 2-fold increased risk of neutropenia in patients with impaired renal function. The most likely chemotherapeutic compound to contribute to this increase in toxicity risk is cyclophosphamide, since the compound and its metabolites are primarily excreted in urine, leading to an increased systemic drug exposure in patients with renal impairment [21]. However, recent guidelines only include recommendations for a dose modification to 75% of the original dose of cyclophosphamide in case of severe renal impairment with GFR 10–29 mL/min and advice against the use of the compound for GFR < 10 mL/min [6]. In the present study, patients with mild renal impairment made up close to three-fourths of the total number of patients with renal impairment and almost no severe renal impairment (~4%) was present in the population. The strong association we identified in this population predominantly consisting of patients with mild renal impairment may indicate that more caution is also warranted for cyclophosphamide administration in this population.

Hepatic impairment was also consistently associated with an increased risk of neutropenia, thrombocytopenia, and anemia. After adjustment for the chemotherapy regimen, hepatic impairment remained associated with anemia. Several chemotherapy regimens were identified that were associated with hematological toxicity in patients with impaired hepatic function. A combination therapy of 30,000 IU bleomycin (day 2, 8 and 15) + 20 mg/m^2^ cisplatin (day 1–5) + 100 mg/m^2^ etoposide (day 1–5) for the treatment of testicular cancer was related to an >2-fold and close to 3.5-fold increased risk of developing thrombocytopenia and anemia, respectively. The most likely contributor to this increased toxicity risk in this treatment regimen is etoposide, which is metabolized in the liver. Current guidelines recommended a dose adjustment to 50% of the original dose of etoposide in case a patient has bilirubin ≥ 50 µmol/L or decreased albumin levels [6]. In the present study almost all (>95%) patients with hepatic impairment were classified as mild given a bilirubin <21 µmol/L, indicating a negative effect in these milder affected patients as well. Monotherapy with melphalan (70–100 mg/m^2^) or methotrexate (1–3000 mg/m^2^) resulted in ~3-fold increased risk of neutropenia and thrombocytopenia. Due to the frequent administration (>70%) of low dose (≤15 mg/m^2^) of methotrexate, effect sizes of high-dosage methotrexate (3000 mg/m^2^) may be heavily diluted. Unfortunately, the effect of high-dosage methotrexate could not be reliably estimated separately because of limited sample size. While for melphalan and methotrexate no dose modification advice is given in current guidelines in case of hepatic impairment, the use of methotrexate is to be avoided in patients with bilirubin ≥86 µmol/L, indicating severe hepatic impairment [6]. Interestingly, patients with hepatic impairment treated with cyclophosphamide were less likely to develop neutropenia and thrombocytopenia during the course of the treatment. These findings can be explained by the prodrug status of cyclophosphamide and the need for metabolization in the liver into active metabolites. The lower incidence of hematological toxicity in patients with hepatic impairment may possibly be an indication of a reduced overall efficacy of cyclophosphamide in this patient population, as we hypothesize that a higher dosage may be needed to compensate for the reduced conversion rate. Currently, cyclophosphamide is contra-indicated for severe hepatic impairment; however, for mild to moderate hepatic impairment, no recommendations are provided [6]. Since the current study consists almost entirely of patients with mild hepatic impairment, the reduced efficacy of cyclophosphamide in current guidelines may be underestimated. Additional analyses are warranted to differentiate between various levels of hepatic impairment in order to determine whether cyclophosphamide can be reliably used in patients with mild to moderate hepatic impairment.

Several limitations regarding the current study should be acknowledged. For renal impairment, we defined impairment based on eGFR solely, in line with current dose modification guidelines. The KDIGO (Kidney Disease: Improving Global Outcomes) guidelines classifying renal impairment using both eGFR-based staging and proteinuria and albuminuria was not a viable option, since these additional parameters are not routinely collected. Also, no consensus exists for evaluating hepatic impairment in the context of dose modification of chemotherapy regimen. In this study, NCI criteria, which rely on total bilirubin and AST levels were used as recommended in oncological settings [13,14]. However, total bilirubin rarely exceeded the threshold of >1.0 ULN. As a result, the classification of hepatic impairment was primarily the result of AST levels. However, AST is a marker of hepatocellular injury rather than hepatic function, and elevations may reflect liver inflammation or damage rather than a reduction in hepatic metabolic capacity or drug clearance. Alternatively, the Child-Pugh score could have been used; however, not all aspects of the score are routinely collected, such as prothrombin time, the presence of ascites, and hepatic encephalopathy. Since total bilirubin and AST levels are routinely collected and commonly used in recommendations for dose modifications, we deemed the NCI criteria most suitable in the current study. However, the optimal classification method for hepatic impairment in an oncological setting by using routinely gathered data, warrants further investigation. Also, the current study was performed with data collected in an specialized university hospital; therefore, results will have to be validated to populations treated in general hospitals. Certain patient groups (i.e., breast cancer patients) may be underrepresented due to referral to closer-by medical specialized care facilities. Furthermore, the risk of hematological toxicity can also be influenced by supportive care strategies including the administration of granulocyte colony-stimulating factor (G-CSF). Correction for such strategies in our analyses was not possible since the use of these strategies was unknown at the time of the analyses. However, we assume that the decision to initiate G-CSF may have lowered the overall incidence of hematologic toxicity, any protective effect would likely have applied equally across groups. Therefore, it is possible that our observed associations are conservative estimates, and the true differences in toxicity risk between patients with and without renal or hepatic impairment may be slightly underestimated. Particularly if G-CSF use was more frequent in the impaired subgroups and prevented progression to grade 3–4 toxicity, which served as our endpoint. To maintain the robustness of the statistical analysis, we included only chemotherapy protocols with a minimum of 10 patients and at least 5 patients with renal or hepatic impairment. Consequently, several protocols were excluded from further analysis. These protocols should be revisited in future multi-center studies, where larger sample sizes will allow for more comprehensive evaluation. Lastly, the size of the study population restricted the number of confounders that could be included in the analysis. Other factors, including cancer type, may influence pharmacokinetics and susceptibility to hematological toxicity. Future large-scale, multi-center studies should evaluate cancer type as a potential confounder.

While the current sample size is limited, these first exploratory analyses of real-world data demonstrate the need for data aggregation across multiple centers and international databases to increase sample size and enable more robust subgroup analyses. Such collaboration is essential to improve evidence-based guidelines on cytotoxic agent dose modification in vulnerable sub-populations. At present, we were able to differentiate normal renal or hepatic functioning from mild to severe impairment, with no power to create additional subpopulations. Therefore, we emphasize that the observed effect may be more prominent with increasing severity of renal or hepatic impairment. On the other hand, if no association has been detected at present, this does not mean that no such effect exists, especially in patients with severely impaired renal or hepatic function. Current guidelines often recommend dose modifications only in cases of severe renal impairment (<30 mL/min GFR). However, the present study identified an increased risk of hematological toxicity across multiple treatment regimens in patients with predominantly mild to moderate renal impairment. Similarly, for hepatic impairment, increased toxicity risk was observed for several compounds despite the study population consisting almost entirely of patients with mild hepatic impairment, where current guidelines typically do not advise dose adjustments. We thus provide insights that may point towards a possible treatment gap in clinical practice.

In order to improve dose modification in the fragile group of patients with impaired renal or hepatic function, the various chemotherapy protocols need to be investigated separately for the risk of hematological toxicity. Administration frequency and time between dosages can influence the proneness of patients for chemotherapy-induced toxicity. While our results demonstrate that overall patients with impaired renal or hepatic function are at increased risk of hematological toxicity, the question always remains whether dose modification should be applied on an individual level because of its impact on treatment efficacy.

## 5. Conclusions

Impaired renal or hepatic function was found to be associated with an increased incidence of hematologic toxicity during treatment with cytotoxic agents. Given that the majority of patients with organ impairment in this cohort had only mild renal or hepatic dysfunction, the observed associations suggest that even lower grades of impairment, which are often not accounted for in current dose modification guidelines, may carry clinical relevance. A more individualized approach to cytotoxic agent dosing, taking into account organ function and compound-specific metabolism and toxicity profiles, is necessary to optimize both safety and efficacy. Multi-center studies of real world data are needed to refine and validate dosing recommendations for patients with varying severity of renal and hepatic impairment.

## Figures and Tables

**Table 1:** Baseline characteristics and follow-up events of patients included in renal impairment analyses.

Baseline Characteristic	Overall *n* = 4489	No Renal Impairment *n* = 4059	Renal Impairment *n* = 430	*p*-Value
Age (years)	62 (53–69)	61 (52–68)	68 (61–74)	<0.001
Male sex (%)	2293 (51%)	2052 (51%)	241 (56%)	0.030
Primary tumor site				<0.001
Breast	170 (3.8%)	160 (3.9%)	10 (2.3%)	
Colorectal/GI	845 (19%)	780 (19%)	65 (15%)	
Genitourinary	291 (6.5%)	220 (5.4%)	71 (17%)	
Gynecological	742 (17%)	690 (17%)	52 (12%)	
Head and neck	872 (19%)	837 (21%)	35 (8.1%)	
Hematological	738 (16%)	618 (15%)	120 (28%)	
Lung	143 (3.2%)	131 (3.2%)	12 (2.8%)	
Other	142 (3.2%)	123 (3.0%)	19 (4.4%)	
Skin	537 (12%)	492 (12%)	45 (10%)	
Unknown	9 (0.2%)	8 (0.2%)	1 (0.2%)	
ALT (U/L)	21 (15–31)	21 (15–32)	19 (13–30)	0.002
AST (U/L)	23 (18–30)	23 (18–30)	22 (17–31)	0.7
Bilirubin (μmol/L)	8 (6–10)	8 (6–10)	7 (6–11)	0.3
eGFR (mL/min)	87 (60–90)	90 (69–90)	48 (35–55)	<0.001
gGT (U/L)	34 (21–64)	35 (21–64)	33 (23–61)	>0.9
Albumin (g/L)	39 (35–42)	39 (35–42)	38 (33–40)	<0.001
Leukocytes, ×10^9^/L	8 (6–10)	8 (6–10)	8 (6–11)	0.081
Neutrophil count, ×10^9^/L	5 (4–7)	5 (4–7)	6 (4–8)	0.002
Platelet count, ×10^9^/L	276 (219–351)	278 (221–351)	260 (203–342)	0.027
Hb (mmol/L)	8 (7–9)	8 (7–9)	7 (6–8)	<0.001
Follow-up events Grade ≥ 3				
Leukopenia	1024 (23%)	900 (22%)	124 (29%)	0.002
Neutropenia	1172 (26%)	1040 (26%)	132 (31%)	0.026
Thrombocytopenia	655 (15%)	571 (14%)	84 (20%)	0.003
Anemia	509 (11%)	431 (11%)	78 (18%)	<0.001

Renal impairment included all categories of severity; mild (*n* = 269), moderate (*n* = 110) to severe (*n* = 51). Data presented as median (interquartile range) or *n* (%). Grade 3–4 hematological toxicity defined as leukopenia (<2.0 × 109/L), neutropenia (<1.0 × 109/L), thrombocytopenia (grade 3–4: <50 × 109/L) and/or anemia (grade 3–4: <4.9 mmol/L). Details on chemotherapy regimen can be found in Supplementary Table S9. ALT = alanine aminotransferase; AST = aspartate transaminase; eGFR = estimated glomerular filtration rate; gGT = gamma-glutamyl transferase; GI = gastrointestinal; Hb = hemoglobulin.

**Table 2:** Baseline characteristics and follow-up events of patients included in hepatic impairment analyses.

Baseline Characteristic	Overall *n* = 6218	No Hepatic Impairment *n* = 4973	Hepatic Impairment *n* = 1245	*p*-Value
Age (years)	60 (50–68)	60 (50–68)	60 (50–67)	0.2
Male sex (%)	3187 (51%)	2595 (52%)	592 (48%)	0.003
Primary tumor site				<0.001
Breast	503 (8.1%)	382 (7.7%)	121 (9.7%)	
Colorectal/GI	1401 (23%)	1043 (21%)	358 (29%)	
Genitourinary	597 (9.6%)	489 (9.8%)	108 (8.7%)	
Gynecological	798 (13%)	662 (13%)	136 (11%)	
Head and neck	944 (15%)	823 (17%)	121 (9.7%)	
Hematological	872 (14%)	684 (14%)	188 (15%)	
Lung	360 (5.8%)	299 (6.0%)	61 (4.9%)	
Other	178 (2.9%)	120 (2.4%)	58 (4.7%)	
Skin	540 (8.7%)	452 (9.1%)	88 (7.1%)	
Unknown	25 (0.4%)	19 (0.4%)	6 (0.5%)	
ALT (U/L)	22 (15–34)	19 (14–27)	43 (27–70)	<0.001
AST (U/L)	23 (18–31)	21 (17–25)	44 (36–62)	<0.001
Bilirubin (μmol/L)	8 (6–11)	8 (6–10)	9 (7–14)	<0.001
eGFR (mL/min)	88 (61–90)	87 (60–90)	90 (67–90)	0.003
gGT (U/L)	36 (22–70)	31 (20–53)	82 (38–203)	<0.001
Albumin (g/L)	39 (35–42)	40 (36–42)	38 (33–42)	<0.001
Leukocytes, ×10^9^/L	8 (6–10)	8 (6–10)	8 (6–10)	0.083
Neutrophil count, ×10^9^/L	5 (4–7)	5 (4–7)	5 (4–7)	0.013
Platelet count, ×10^9^/L	275 (220–348)	275 (222–345)	273 (208–356)	0.13
Hb (mmol/L)	8 (7–9)	8 (8–9)	8 (7–9)	<0.001
Follow-up events Grade ≥ 3				
Leukopenia	1623 (26%)	1259 (25%)	364 (29%)	0.005
Neutropenia	2090 (34%)	1609 (32%)	481 (39%)	<0.001
Thrombocytopenia	906 (15%)	683 (14%)	223 (18%)	<0.001
Anemia	714 (11%)	519 (10%)	195 (16%)	<0.001

Hepatic impairment included all categories of severity; mild (*n* = 1186), moderate (*n* = 44), and severe (*n* = 15). Data presented as median (interquartile range) or *n* (%). Details on chemotherapy regimen can be found in Supplementary Table S10. ALT = alanine aminotransferase; AST = aspartate transaminase; eGFR = estimated glomerular filtration rate; gGT = gamma-glutamyl transferase; Hb = hemoglobulin.

**Table 3:** Unadjusted and adjusted hazard ratios of mild to severely impaired renal function versus normal renal function in association with hematological toxicity.

	Neutropenia	Thrombocytopenia	Anemia
Model 1	1.43 [1.18–1.73]	1.46 [1.15–1.86]	1.66 [1.27–2.16]
Model 2	1.54 [1.27–1.88]	1.51 [1.19–1.92]	1.86 [1.42–2.43]
Model 3	1.26 [1.03–1.55]	1.02 [0.79–1.32]	1.51 [1.15–1.99]
Model 4	1.44 [1.13–1.84]	1.19 [0.87–1.63]	1.75 [1.24–2.46]

Mild to severely impaired renal functioning was defined as an glomerular filtration rate (GFR) (CKD-EPI) of <60 mL/min. Hematological toxicities were defined as grade 3–4. Model 1 consisted of the unadjusted cox regression analysis. Model 2 consisted of the cox regression analyses adjusted for age and sex. Model 3 presents of the cox regression analysis adjusted for the chemotherapy protocol. Model 4 contains the results of the cox regression analysis adjusted for the chemotherapy protocol in a patient subset treated with a predetermined short-list of compounds metabolized by the kidney and therefore likely associated with toxicity in patients with renal impairment. Results are presented as hazard ratio (HR) with [95% CI].

**Table 4:** Unadjusted hazard ratios of impaired renal function (<60 mL/min GFR) in relation to hematological toxicity per chemotherapy regimen.

Chemotherapy Regimen	*n*	Neutropenia	*n*	Thrombocytopenia	*n*	Anemia	*n*
Bortezomib + cyclophosphamide	26	0.75 [0.30–1.92]	18	0.67 [0.26–1.76]	18	0.79 [0.21–3.00]	9
Carboplatin + gemcitabine	50	1.90 [0.76–4.74]	29	0.85 [0.28–2.57]	20	1.14 [0.44–3.00]	20
Carboplatin + paclitaxel	706	1.30 [0.79–1.87]	256	1.10 [0.50–2.40]	71	1.06 [0.48–2.32]	69
Cyclophosphamide	198	1.06 [0.55–2.07]	77	1.07 [0.55–2.09]	71	1.11 [0.43–2.86]	35
Cyclophosphamide + doxorubicin	128	2.18 [0.93–5.11]	80	NA	23	5.57 [1.88–16.46] **	18
Cyclophosphamide + doxorubicin + rituximab + vincristine	182	1.86 [1.11–3.11] *	128	1.38 [0.62–3.08]	47	2.53 [1.20–5.34] *	38
Melphalan	152	1.46 [0.66–3.24]	53	1.54 [0.69–3.41]	51	NA	14
Mitomycin	133	NA	12	NA	4	2.48 [0.94–6.54]	30

Numbers (*n*) presented are the total number of individual patients receiving the chemotherapy compound or combination therapy, and the total number of patients experiencing the outcome of interest, i.e., neutropenia, thrombocytopenia, and anemia. The hazard ratio [95% CI] represents the risk of hematologic toxicity for patients with impaired renal function versus normal renal function. A minimum of 5 events of the outcome of interest were needed in both normal and impaired renal functioning categories, otherwise “NA” is indicated. Detailed descriptives per analysis are provided in Supplementary B.1. *p* values are indicated by asterixis as follows: * *p* value <0.05, ** *p* value <0.01.

**Table 5:** Unadjusted and adjusted hazard ratios of mild to severely impaired hepatic function versus normal hepatic function in association with hematological toxicity.

	Neutropenia	Thrombocytopenia	Anemia
Model 1	1.25 [1.11–1.40]	1.33 [1.13–1.57]	1.62 [1.34–1.95]
Model 2	1.27 [1.13–1.43]	1.38 [1.17–1.63]	1.61 [1.34–1.94]
Model 3	1.10 [0.98–1.25]	1.15 [0.97–1.36]	1.33 [1.10–1.61]
Model 4	1.14 [1.00–1.31]	1.20 [0.98–1.47]	1.34 [1.06–1.69]

Model 1 consisted of the unadjusted cox regression analysis. Model 2 presents the cox regression analysis adjusted for age and sex. Model 3 consisted of the cox regression analysis adjusted for the chemotherapy regimen. Model 4 presents the results of the cox regression analysis adjusted for the chemotherapy regimen in a patient subset treated with a predetermined short-list of high-priority compounds. Results are presented as hazard ratio (HR) with [95% CI].

**Table 6:** Unadjusted hazard ratios of impaired hepatic function in relation to hematological toxicity per chemotherapy regimen.

Chemotherapy Regimen	*n*	Neutropenia	*n*	Thrombocytopenia	*n*	Anemia	*n*
Bevacizumab + fluorouracil + irinotecan + oxaliplatin	38	1.81 [0.43–7.65]	18	NA	1	NA	4
Bleomycin + cisplatin + etoposide	236	1.18 [0.76–1.83]	210	2.38 [1.08–5.20] *	27	3.46 [1.29–9.33] *	16
Bortezomib	116	1.26 [0.65–2.43]	59	1.06 [0.57–1.99]	65	1.63 [0.73–3.66]	31
Capecitabine + cisplatin + epirubicin	85	1.01 [0.23–4.46]	35	NA	4	NA	3
Carboplatin + etoposide	47	0.97 [0.37–2.50]	34	1.14 [0.48–2.69]	24	1.58 [0.51–4.86]	13
Carboplatin + paclitaxel	706	0.91 [0.63–1.31]	256	0.73 [0.38–1.41]	71	1.42 [0.82–2.45]	69
Carboplatin + pemetrexed	66	1.63 [0.54–4.96]	26	NA	16	NA	11
Carmustine + methotrexate + teniposide	16	3.09 [0.94–10.10]	15	NA	8	NA	5
Cisplatin + etoposide	152	1.44 [0.88–2.36]	135	1.06 [0.44–2.58]	34	0.16 [0.02–1.21]	28
Cisplatin + gemcitabine	131	1.27 [0.59–2.73]	63	1.39 [0.66–2.93]	29	2.21 [0.87–5.59]	18
Cyclophosphamide	198	0.30 [0.13–0.69] **	77	0.32 [0.14–0.74] **	71	0.54 [0.21–1.39]	35
Cyclophosphamide + docetaxel + epirubicin + fluorouracil	202	0.94 [0.50–1.77]	122	NA	9	NA	5
Cyclophosphamide + doxorubicin	128	1.02 [0.57–1.83]	80	1.15 [0.44–2.98]	23	2.28 [0.87–6.01]	18
Cyclophosphamide + doxorubicin + rituximab + vincristine	182	1.39 [0.88–2.18]	128	1.57 [0.86–2.88]	47	1.49 [0.76–2.91]	38
Cyclophosphamide + fludarabine	47	1.05 [0.53–2.09]	46	1.72 [0.82–3.63]	35	1.98 [0.87–4.50]	26
Doxorubicin	38	1.99 [0.66–6.05]	16	NA	4	15.06 [1.81–125.42] *	7
Fluorouracil + irinotecan + oxaliplatin	53	1.32 [0.43–4.05]	20	NA	3	NA	3
Gemcitabine	58	1.73 [0.57–5.25]	37	NA	6	NA	6
Melphalan	152	2.91 [1.42–5.98] **	53	3.07 [1.49–6.31] **	51	NA	14
Methotrexate	76	3.05 [1.57–5.91] ***	44	2.98 [1.39–6.41] **	35	2.02 [0.87–4.71]	30
Paclitaxel	61	1.99 [0.65–6.11]	16	NA	2	NA	6

Numbers (*n*) presented are the total number of individual patients receiving the chemotherapy compound or combination therapy, and the total number of patients experiencing the outcome of interest, i.e., neutropenia, thrombocytopenia, and anemia. The hazard ratio [95% CI] represents the risk of hematologic toxicity for patients with impaired hepatic function versus normal hepatic function. A minimum of 5 events of the outcome of interest were needed in both normal and impaired hepatic functioning categories, otherwise “NA” is indicated. Detailed descriptives per analysis are provided in Supplementary B.2. *p* values are indicated by asterixis as follows: * *p* value <0.05, ** *p* value <0.01, *** *p* value <0.001.

## Data Availability

The datasets presented in this article are not readily available due to the inclusion of pseudonymized patient data containing personal information governed by the General Data Protection Regulation (GDPR). Reasonable requests for data access in the context of scientific collaboration can be directed to the corresponding author.

## Supplementary Materials

The following supporting information can be downloaded at: https://www.mdpi.com/article/10.3390/pharmaceutics17101280/s1, Supplementary A: Table S1: Compounds of interest for impaired renal functioning; Table S2: Compounds of interest for impaired hepatic functioning; Table S3: Baseline characteristics of the patients included in the survival analyses of impaired renal function in relation to grade ≥ 3 neutropenia; Table S4: Baseline characteristics of the patients included in the survival analyses of impaired renal function in relation to grade ≥ 3 thrombocytopenia; Table S5: Baseline characteristics of the patients included in the survival analyses of impaired renal function in relation to grade ≥ 3 anemia; Table S6: Baseline characteristics of the patients included in the survival analyses of impaired hepatic function in relation to grade ≥ 3 neutropenia; Table S7: Baseline characteristics of the patients included in the survival analyses of impaired hepatic function in relation to grade ≥ 3 thrombocytopenia; Table S8: Baseline characteristics of the patients included in the survival analyses of impaired hepatic function in relation to grade ≥ 3 anemia; Table S9: Extended table of baseline characteristics and follow-up events of patients included in renal impairment analyses including the administered chemotherapeutic compounds; Table S10: Extended table of baseline characteristics and follow-up events of patients included in hepatic impairment analyses including the administered chemotherapeutic compounds; Table S11: Baseline characteristics and follow-up events of the subset of patients included in renal impairment analyses that were treated with a compound from a pre-selected list of higher-risk compounds involved in renal clearance; Table S12: Unadjusted and adjusted hazard ratios of impaired renal function categorized by severity in relation to hematological toxicity; Table S13: Baseline characteristics and follow-up events of the subset of patients included in hepatic impairment analyses that were treated with a compound from a pre-selected list of higher-risk compounds involved in hepatic metabolism; Table S14: Incidence of hematological toxicity during chemotherapy regimen compared between organ impairment subgroups. Supplementary B: Overview of baseline characteristics and hematological toxicity events per chemotherapeutic regimen. Table S15: Baseline characteristics and follow-up events for patients with and without renal impairment receiving bortezomib + cyclophosphamide; Table S16: Baseline characteristics and follow-up events for patients with and without renal impairment receiving carboplatin + gemcitabine; Table S17: Baseline characteristics and follow-up events for patients with and without renal impairment receiving carboplatin + paclitaxel; Table S18: Baseline characteristics and follow-up events for patients with and without renal impairment receiving cyclophosphamide; Table S19: Baseline characteristics and follow-up events for patients with and without renal impairment receiving cyclophosphamide + doxorubicin; Table S20: Baseline characteristics and follow-up events for patients with and without renal impairment receiving cyclophosphamide + doxorubicin + rituximab + vincristine; Table S21: Baseline characteristics and follow-up events for patients with and without renal impairment receiving melphalan; Table S22: Baseline characteristics and follow-up events for patients with and without renal impairment receiving mitomycin. Table S23: Baseline characteristics and follow-up events for patients with and without hepatic impairment receiving bevacizumab + fluorouracil + irinotecan + oxaliplatin; Table S24: Baseline characteristics and follow-up events for patients with and without hepatic impairment receiving bleomycin + cisplatin + etoposide; Table S25: Baseline characteristics and follow-up events for patients with and without hepatic impairment receiving bortezomib; Table S26: Baseline characteristics and follow-up events for patients with and without hepatic impairment receiving capecitabine + cisplatin + epirubicin; Table S27: Baseline characteristics and follow-up events for patients with and without hepatic impairment receiving carboplatin + etoposide; Table S28: Baseline characteristics and follow-up events for patients with and without hepatic impairment receiving carboplatin + paclitaxel; Table S29: Baseline characteristics and follow-up events for patients with and without hepatic impairment receiving carboplatin + pemetrexed; Table S30: Baseline characteristics and follow-up events for patients with and without hepatic impairment receiving carmustine + methotrexate + teniposide; Table S31: Baseline characteristics and follow-up events for patients with and without hepatic impairment receiving cisplatin + etoposide; Table S32: Baseline characteristics and follow-up events for patients with and without hepatic impairment receiving cisplatin + gemcitabine; Table S33: Baseline characteristics and follow-up events for patients with and without hepatic impairment receiving cyclophosphamide; Table S34: Baseline characteristics and follow-up events for patients with and without hepatic impairment receiving cyclophosphamide + docetaxel + epirubicin + fluorouracil; Table S35: Baseline characteristics and follow-up events for patients with and without hepatic impairment receiving cyclophosphamide + doxorubicin; Table S36: Baseline characteristics and follow-up events for patients with and without hepatic impairment receiving cyclophosphamide + doxorubicin + rituximab + vincristine; Table S37: Baseline characteristics and follow-up events for patients with and without hepatic impairment receiving cyclophosphamide + fludarabine; Table S38: Baseline characteristics and follow-up events for patients with and without hepatic impairment receiving doxorubicin; Table S39: Baseline characteristics and follow-up events for patients with and without hepatic impairment receiving fluorouracil + irinotecan + oxaliplatin; Table S40: Baseline characteristics and follow-up events for patients with and without hepatic impairment receiving gemcitabine; Table S41: Baseline characteristics and follow-up events for patients with and without hepatic impairment receiving melphalan; Table S42: Baseline characteristics and follow-up events for patients with and without hepatic impairment receiving methotrexate; Table S43: Baseline characteristics and follow-up events for patients with and without hepatic impairment receiving paclitaxel. Supplementary C: Supplementary excel file containing an overview of hematological toxicity event rates per chemotherapy regimen and grouped by sex, or renal/hepatic impairment.

## Abbreviations

The following abbreviations are used in this manuscript:

ALT	Alanine Aminotransferase
AST	Aspartate Aminotransferase
CKD-EPI	Chronic Kidney Disease Epidemiology Collaboration
CTCAE	Common Terminology Criteria for Adverse Events
GFR	Glomerular Filtration Rate
Hb	Hemoglobin
HR	Hazard Ratio
ULN	Upper Limit of Normal
UMCU	University Medical Centre Utrecht
UPOD	Utrecht Patient Oriented Database
