# Supplementary material for: Chemotherapy-Induced Hematological Toxicity in Patients with Renal or Hepatic Impairment
Source: Pharmaceutics. 2025 Sep 30;17(10):1280. doi: 10.3390/pharmaceutics17101280 (PMC12567060; doi:10.3390/pharmaceutics17101280)
Supplement: Supplementary file 1 [file pharmaceutics-17-01280-s001.zip › Supplementary S1 and S2.pdf]

Supplementary Materials

Supplementary S1

Table S1. Compounds of interest for impaired renal functioning.

Included Compounds	Excluded Compounds
amsacrine (L01XX01)	atezolizumab (L01FF05)
arsenic trioxide (L01XX27)	avelumab (L01FF04)
bleomycin (L01DC01)	azacitidine (L01BC07)
brentuximab vedotin (L01FX05)	bendamustine (L01AA09)
capecitabine (L01BC06)	bevacizumab (L01FG01)
carboplatin (L01XA02)	blinatumomab (L01FX07)
carmustine (L01AD01)	bortezomib (L01XG01)
cisplatin (L01XA01)	busulfan (L01AB01)
cladribine (L01BB04)	cabazitaxel (L01CD04)
cyclophosphamide (L01AA01)	carfilzomib (L01XG02)
cytarabine (L01BC01)	cemiplimab (L01FF06)
dacarbazine (L01AX04)	cetuximab (L01FE01)
daunorubicin (L01DB02)	chlorambucil (L01AA02)
eribuline (L01XX41)	dactinomycin (L01DA01)
etoposide (L01CB01)	daratumumab (L01FC01)
fluorouracil (L01BC02)	docetaxel (L01CD02)
idarubicine (L01DB06)	dostarlimab (L01FF07)
ifosfamide (L01AA06)	doxorubicin (L01DB01)
irinotecan (L01CE02)	doxorubicin liposomal (L01DB01)
irinotecan liposomal (L01CE02)	durvalumab (L01FF03)
lomustine (L01AD02)	enfortumab vedotin (L01FX13)
melphalan (L01AA03)	epirubicin (L01DB03)
mercaptopurine (L01BB02)	fludarabine (L01BB05)
methotrexate (L01BA01)	gemcitabine (L01BC05)
mitomycine (L01DC03)	inotuzumab (L01FB01)
mitoxantrone (L01DB07)	ipilimumab (L01FX04)
oxaliplatine (L01XA03)	nivolumab (L01FF01)
pembrolizumab (L01FF02)	obinutuzumab (L01FA03)
pemetrexed (L01BA04)	paclitaxel (L01CD01)
polatuzumab vedotin (L01FX14)	panitumumab (L01FE02)
teniposide (L01CB02)	pazopanib (L01EX03)
thiotepa (L01AC01)	pegaspargase (L01XX24)
topotecan (L01CE01)	pertuzumab (L01FD02)
	procarbazine (L01XB01)
	ramucirumab (L01FG02)
	rituximab (L01FA01)
	thioguanine (L01BB03)
	trastuzumab (L01FD01)
	tremelimumab (L01FX20)
	treosulfan (L01AB02)
	tretinoin (L01XF01)
	vinblastine (L01CA01)
	vincristine (L01CA02)
	vinorelbine (L01CA04)

Table S2. Compounds of interest for impaired hepatic functioning.

Included Compounds	Excluded Compounds
amsacrine (L01XX01)	atezolizumab (L01FF05)
arsenic trioxide (L01XX27)	avelumab (L01FF04)
azacitidine (L01BC07)	bevacizumab (L01FG01)
bendamustine (L01AA09)	bleomycin (L01DC01)
bortezomib (L01XG01)	blinatumomab (L01FX07)
brentuximab vedotin (L01FX05)	busulfan (L01AB01)
cabazitaxel (L01CD04)	capecitabine (L01BC06)
carfilzomib (L01XG02)	carboplatin (L01XA02)
carmustine (L01AD01)	cemiplimab (L01FF06)
cladribine (L01BB04)	cetuximab (L01FE01)
cyclophosphamide (L01AA01)	chlorambucil (L01AA02)
cytarabine (L01BC01)	cisplatin (L01XA01)
dacarbazine (L01AX04)	dactinomycin (L01DA01)
daunorubicin (L01DB02)	daratumumab (L01FC01)
docetaxel (L01CD02)	dostarlimab (L01FF07)
doxorubicin (L01DB01)	durvalumab (L01FF03)
doxorubicin liposomal (L01DB01)	enfortumab vedotin (L01FX13)
epirubicin (L01DB03)	fludarabine (L01BB05)
eribulin (L01XX41)	ifosfamide (L01AA06)
etoposide (L01CB01)	inotuzumab (L01FB01)
fluorouracil (L01BC02)	ipilimumab (L01FX04)
gemcitabine (L01BC05)	nivolumab (L01FF01)
idarubicine (L01DB06)	obinutuzumab (L01FA03)
irinotecan (L01CE02)	oxaliplatine (L01XA03)
irinotecan liposomal (L01CE02)	panitumumab (L01FE02)
melphalan (L01AA03)	pegaspargase (L01XX24)
mercaptopurine (L01BB02)	pembrolizumab (L01FF02)
methotrexate (L01BA01)	pertuzumab (L01FD02)
mitomycine (L01DC03)	procarbazine (L01XB01)
mitoxantrone (L01DB07)	ramucirumab (L01FG02)
paclitaxel (L01CD01)	rituximab (L01FA01)
pazopanib (L01EX03)	thioguanine (L01BB03)
pemetrexed (L01BA04)	trastuzumab (L01FD01)
polatuzumab vedotin (L01FX14)	tremelimumab (L01FX20)
teniposide (L01CB02)	treosulfan (L01AB02)
thiotepa (L01AC01)	tretinoin (L01XF01)
topotecan (L01CE01)	
vinblastine (L01CA01)	
vincristine (L01CA02)	
vinorelbine (L01CA04)	

Table S3. Baseline characteristics of the patients included in the survival analyses of impaired renal function in relation to grade ≥ 3 neutropenia.

Baseline Characteristic	Overall<br>n = 3812	No Renal Impairment<br>n = 3468	Renal Impairment<br>n = 344	p-Value
Age (years)	61 (52–68)	61 (52–68)	67 (59–73)	<0.001
Male sex (%)	1863 (49%)	1681 (48%)	182 (53%)	0.12
ALT (U/L)	21 (15–31)	21 (15–32)	19 (13–28)	<0.001
AST (U/L)	22 (18–30)	22 (18–30)	22 (17–30)	0.5
Bilirubin (μmol/L)	8 (6–11)	8 (6–11)	8 (6–11)	0.6
eGFR (ml/min)	87 (60–90)	90 (71–90)	49 (37–55)	<0.001
gGT (U/L)	35 (21–66)	35 (21–67)	34 (23–63)	0.8
Albumin (g/L)	39 (35–42)	39 (35–42)	37 (33–41)	<0.001
Leukocytes, ×10^9/L	8 (6–10)	8 (6–10)	8 (6–11)	0.021
Neutrophil count, ×10^9/L	5 (4–7)	5 (4–7)	6 (4–8)	<0.001
Platelet count, ×10^9/L	276 (221–350)	277 (223–350)	263 (206–339)	0.030
Hb (mmol/L)	8 (7–9)	8 (7–9)	7 (6–8)	<0.001
**Follow-up events**				
Leukopenia	1005 (26%)	884 (25%)	121 (35%)	<0.001
Neutropenia	1148 (30%)	1019 (29%)	129 (38%)	0.002
Thrombocytopenia	639 (17%)	556 (16%)	83 (24%)	<0.001
Anemia	480 (13%)	409 (12%)	71 (21%)	<0.001

Data presented as median (interquartile range) or n (%). ALT = alanine aminotransferase; AST = aspartate transaminase; eGFR = estimated glomerular filtration rate; gGT = gamma-glutamyl transferase; Hb = hemoglobulin.

Table S4. Baseline characteristics of the patients included in the survival analyses of impaired renal function in relation to grade ≥ 3 thrombocytopenia.

Baseline Characteristic	Overall<br>n = 2890	No Renal Impairment<br>n = 2642	Renal Impairment<br>n = 248	p-Value
Age (years)	60 (51–67)	60 (51–67)	66 (58–71)	<0.001
Male sex (%)	1315 (46%)	1195 (45%)	120 (48%)	0.3
ALT (U/L)	21 (15–31)	21 (15–31)	19 (13–31)	0.031
AST (U/L)	22 (18–29)	22 (18–29)	22 (17–33)	0.6
Bilirubin (μmol/L)	8 (6–10)	8 (6–10)	8 (6–10)	0.4
eGFR (ml/min)	87 (60–90)	90 (69–90)	47 (35–55)	<0.001
gGT (U/L)	33 (21–63)	33 (20–63)	33 (23–63)	0.6
Albumin (g/L)	39 (35–42)	39 (35–42)	38 (32–40)	<0.001
Leukocytes, ×10^9/L	8 (6–10)	8 (6–10)	8 (6–11)	0.11
Neutrophil count, ×10^9/L	5 (4–7)	5 (4–7)	6 (4–8)	0.004
Platelet count, ×10^9/L	279 (221–352)	281 (222–353)	262 (203–345)	0.045
Hb (mmol/L)	8 (7–9)	8 (7–9)	7 (6–8)	<0.001
**Follow-up events**				
Leukopenia	943 (33%)	834 (32%)	109 (44%)	<0.001
Neutropenia	1056 (37%)	943 (36%)	113 (46%)	0.003
Thrombocytopenia	607 (21%)	529 (20%)	78 (31%)	<0.001
Anemia	416 (14%)	357 (14%)	59 (24%)	<0.001

Data presented as median (interquartile range) or n (%). ALT = alanine aminotransferase; AST = aspartate transaminase; eGFR = estimated glomerular filtration rate; gGT = gamma-glutamyl transferase; Hb = hemoglobulin.

Table S5. Baseline characteristics of the patients included in the survival analyses of impaired renal function in relation to grade ≥ 3 anemia.

Baseline Characteristic	Overall<br>n = 3594	No Renal Impairment<br>n = 3280	Renal Impairment<br>n = 314	p-Value
Age (years)	61 (52–68)	60 (51–68)	67 (59–73)	<0.001
Male sex (%)	1736 (48%)	1568 (48%)	168 (54%)	0.054
ALT (U/L)	21 (15–31)	21 (15–31)	19 (14–28)	0.006
AST (U/L)	22 (17–29)	22 (17–29)	22 (17–28)	0.4
Bilirubin (μmol/L)	8 (6–11)	8 (6–11)	8 (6–11)	0.4
eGFR (ml/min)	88 (64–90)	90 (73–90)	49 (39–55)	<0.001
gGT (U/L)	34 (21–65)	34 (21–65)	34 (23–63)	0.5
Albumin (g/L)	39 (35–42)	39 (35–42)	38 (33–41)	0.002
Leukocytes, $\times 10^9$/L	8 (6–10)	8 (6–10)	8 (6–10)	0.2
Neutrophil count, $\times 10^9$/L	5 (4–7)	5 (4–7)	6 (4–7)	0.011
Platelet count, $\times 10^9$/L	280 (224–352)	282 (225–354)	265 (206–340)	0.010
Hb (mmol/L)	8 (7–9)	8 (7–9)	8 (6–9)	<0.001
**Follow-up events**				
Leukopenia	940 (26%)	837 (26%)	103 (33%)	0.006
Neutropenia	1059 (30%)	952 (29%)	107 (35%)	0.070
Thrombocytopenia	596 (17%)	525 (16%)	71 (23%)	0.003
Anemia	457 (13%)	394 (12%)	63 (20%)	<0.001

Data presented as median (interquartile range) or n (%). ALT = alanine aminotransferase; AST = aspartate transaminase; eGFR = estimated glomerular filtration rate; gGT = gamma-glutamyl transferase; Hb = hemoglobulin.

Table S6. Baseline characteristics of the patients included in the survival analyses of impaired hepatic function in relation to grade ≥ 3 neutropenia.

Baseline Characteristic	Overall<br>n = 5247	No Hepatic Impairment<br>n = 4276	Hepatic Impairment<br>n = 971	p-Value
Age (years)	60 (49–67)	60 (49–67)	59 (50–67)	0.8
Male sex (%)	2603 (50%)	2162 (51%)	441 (45%)	0.004
ALT (U/L)	21 (15–32)	19 (14–27)	42 (26–69)	<0.001
AST (U/L)	23 (18–30)	21 (17–25)	43 (36–62)	<0.001
Bilirubin (μmol/L)	8 (6–11)	8 (6–10)	9 (7–13)	<0.001
eGFR (ml/min)	88 (60–90)	87 (60–90)	89 (62–90)	0.067
gGT (U/L)	35 (21–67)	31 (20–52)	72 (35–175)	<0.001
Albumin (g/L)	39 (35–42)	40 (36–42)	38 (34–42)	<0.001
Leukocytes, $\times 10^9$/L	8 (6–10)	8 (6–10)	8 (6–10)	0.064
Neutrophil count, $\times 10^9$/L	5 (4–7)	5 (4–7)	5 (4–7)	<0.001
Platelet count, $\times 10^9$/L	274 (219–345)	274 (222–343)	269 (207–351)	0.062
Hb (mmol/L)	8 (7–9)	8 (8–9)	8 (7–9)	<0.001
**Follow-up events**				
Leukopenia	1564 (30%)	1227 (29%)	337 (35%)	<0.001
Neutropenia	2006 (38%)	1565 (37%)	441 (45%)	<0.001
Thrombocytopenia	878 (17%)	667 (16%)	211 (22%)	<0.001
Anemia	669 (13%)	490 (11%)	179 (18%)	<0.001

Data presented as median (interquartile range) or n (%). ALT = alanine aminotransferase; AST = aspartate transaminase; eGFR = estimated glomerular filtration rate; gGT = gamma-glutamyl transferase; Hb = hemoglobulin.

Table S7. Baseline characteristics of the patients included in the survival analyses of impaired hepatic function in relation to grade ≥ 3 thrombocytopenia.

Baseline Characteristic	Overall n = 3508	No Hepatic Impairment n = 2897	Hepatic Impairment n = 611	p-Value
Age (years)	59 (48–67)	59 (48–67)	59 (48–67)	>0.9
Male sex (%)	1774 (51%)	1494 (52%)	280 (46%)	0.010
ALT (U/L)	21 (15–32)	19 (14–26)	43 (27–70)	<0.001
AST (U/L)	23 (18–30)	21 (17–25)	43 (36–61)	<0.001
Bilirubin (μmol/L)	8 (6–11)	8 (6–10)	9 (7–13)	<0.001
eGFR (ml/min)	89 (60–90)	88 (60–90)	90 (63–90)	0.3
gGT (U/L)	33 (21–62)	30 (19–50)	67 (33–150)	<0.001
Albumin (g/L)	39 (35–42)	40 (36–42)	38 (34–42)	0.002
Leukocytes, $\times 10^9$/L	8 (6–10)	8 (6–10)	8 (6–11)	0.2
Neutrophil count, $\times 10^9$/L	5 (4–7)	5 (4–7)	5 (4–8)	0.055
Platelet count, $\times 10^9$/L	278 (221–351)	279 (223–351)	269 (205–351)	0.022
Hb (mmol/L)	8 (7–9)	8 (7–9)	8 (7–9)	<0.001
**Follow-up events**				
Leukopenia	1269 (36%)	1001 (35%)	268 (44%)	<0.001
Neutropenia	1553 (44%)	1218 (42%)	335 (55%)	<0.001
Thrombocytopenia	779 (22%)	596 (21%)	183 (30%)	<0.001
Anemia	539 (15%)	395 (14%)	144 (24%)	<0.001

Data presented as median (interquartile range) or *n* (%). ALT = alanine aminotransferase; AST = aspartate transaminase; eGFR = estimated glomerular filtration rate; gGT = gamma-glutamyl transferase; Hb = hemoglobulin.

Table S8. Baseline characteristics of the patients included in the survival analyses of impaired hepatic function in relation to grade ≥ 3 anemia.

Baseline Characteristic	Overall n = 4188	No Hepatic Impairment n = 3469	Hepatic Impairment n = 719	p-Value
Age (years)	60 (49–68)	60 (49–68)	59 (48–67)	0.15
Male sex (%)	2204 (52%)	1852 (53%)	352 (48%)	0.021
ALT (U/L)	21 (15–32)	19 (14–26)	43 (27–70)	<0.001
AST (U/L)	22 (18–29)	21 (16–25)	43 (36–62)	<0.001
Bilirubin (μmol/L)	8 (6–11)	8 (6–10)	9 (6–13)	<0.001
eGFR (ml/min)	89 (65–90)	88 (63–90)	90 (69–90)	0.026
gGT (U/L)	34 (21–64)	30 (20–50)	71 (34–167)	<0.001
Albumin (g/L)	39 (35–42)	39 (36–42)	38 (33–42)	<0.001
Leukocytes, $\times 10^9$/L	8 (6–10)	8 (6–10)	8 (6–11)	0.044
Neutrophil count, $\times 10^9$/L	5 (4–7)	5 (4–7)	5 (4–8)	0.008
Platelet count, $\times 10^9$/L	278 (223–351)	278 (225–350)	277 (211–359)	0.3
Hb (mmol/L)	8 (7–9)	8 (8–9)	8 (7–9)	<0.001
**Follow-up events**				
Leukopenia	1264 (30%)	998 (29%)	266 (37%)	<0.001
Neutropenia	1554 (37%)	1223 (35%)	331 (46%)	<0.001
Thrombocytopenia	767 (18%)	587 (17%)	180 (25%)	<0.001
Anemia	584 (14%)	433 (12%)	151 (21%)	<0.001

Data presented as median (interquartile range) or *n* (%). ALT = alanine aminotransferase; AST = aspartate transaminase; eGFR = estimated glomerular filtration rate; gGT = gamma-glutamyl transferase; Hb = hemoglobulin.

Table S9. Extended table of baseline characteristics and follow-up events of patients included in renal impairment analyses including the administered chemotherapeutic compounds.

Baseline Characteristic	Overall n = 4489	No Renal Impairment n = 4059	Renal Impairment n = 430	p-Value
Age (years)	62 (53–69)	61 (52–68)	68 (61–74)	<0.001
Male sex (%)	2293 (51%)	2052 (51%)	241 (56%)	0.030
Compound				
Bevacizumab	131 (2.9%)	126 (3.1%)	5 (1.2%)	
Bortezomib	116 (2.6%)	72 (1.8%)	44 (10%)	
Bortezomib + cyclophosphamide	26 (0.6%)	14 (0.3%)	12 (2.8%)	
Busulfan + fludarabine	152 (3.4%)	143 (3.5%)	9 (2.1%)	
Capecitabine + oxaliplatin	174 (3.9%)	166 (4.1%)	8 (1.9%)	
Carboplatin	59 (1.3%)	50 (1.2%)	9 (2.1%)	
Carboplatin + gemcitabine	50 (1.1%)	39 (1.0%)	11 (2.6%)	
Carboplatin + paclitaxel	706 (16%)	639 (16%)	67 (16%)	
Cetuximab	125 (2.8%)	103 (2.5%)	22 (5.1%)	
Cisplatin	990 (22%)	972 (24%)	18 (4.2%)	
Cisplatin + gemcitabine	131 (2.9%)	126 (3.1%)	5 (1.2%)	
Cyclophosphamide	198 (4.4%)	171 (4.2%)	27 (6.3%)	
Cyclophosphamide + doxorubicin	128 (2.9%)	120 (3.0%)	8 (1.9%)	
Cyclophosphamide + doxorubicin + rituximab +vincristine	182 (4.1%)	160 (3.9%)	22 (5.1%)	
docetaxel	103 (2.3%)	88 (2.2%)	15 (3.5%)	
Ipilimumab + nivolumab	262 (5.8%)	228 (5.6%)	34 (7.9%)	
Melphalan	152 (3.4%)	136 (3.4%)	16 (3.7%)	
Mitomycin	133 (3.0%)	121 (3.0%)	12 (2.8%)	
Nivolumab	159 (3.5%)	134 (3.3%)	25 (5.8%)	
Paclitaxel	61 (1.4%)	50 (1.2%)	11 (2.6%)	
Pembrolizumab	394 (8.8%)	353 (8.7%)	41 (9.5%)	
Rituximab	57 (1.3%)	48 (1.2%)	9 (2.1%)	
Lab values				
ALT (U/L)	21 (15–32)	21 (15–32)	19 (13–26)	<0.001
AST (U/L)	22 (18–30)	22 (18–30)	21 (17–28)	0.057
Bilirubin (μmol/L)	8 (6–11)	8 (6–11)	8 (6–10)	0.066
eGFR (ml/min)	87 (62–90)	90 (72–90)	49 (39–55)	<0.001
gGT (U/L)	35 (21–66)	35 (21–67)	35 (24–63)	0.4
Albumin (g/L)	39 (35–42)	39 (35–42)	38 (34–41)	<0.001
Leukocytes,$\times 10^9$/L	8 (6–10)	8 (6–10)	8 (7–11)	0.009
Neutrophil count, $\times 10^9$/L	5 (4–7)	5 (4–7)	6 (4–8)	<0.001
Platelet count,$\times 10^9$/L	277 (222–351)	278 (223–351)	266 (213–349)	0.2
Hb (mmol/L)	8 (7–9)	8 (7–9)	8 (7–8)	<0.001
**Follow-up events**				
Leukopenia	1024 (23%)	900 (22%)	124 (29%)	0.002
Neutropenia	1172 (26%)	1040 (26%)	132 (31%)	0.026
Thrombocytopenia	655 (15%)	571 (14%)	84 (20%)	0.003
Anemia	509 (11%)	431 (11%)	78 (18%)	<0.001

Data presented as median (interquartile range) or *n* (%). Grade 3–4 hematological toxicity defined as leukopenia (<2.0 × 10^9/L), neutropenia (<1.0 × 10^9/L), thrombocytopenia (grade 3–4: <50 × 10^9/L) and/or anemia (grade 3–4: <4.9 mmol/L). ALT = alanine aminotransferase; AST = aspartate transaminase; eGFR = estimated glomerular filtration rate; gGT = gamma-glutamyl transferase; Hb = hemoglobulin.

Table S10. Extended table of baseline characteristics and follow-up events of patients included in hepatic impairment analyses including the administered chemotherapeutic compounds.

Baseline Characteristic	Overall *n* = 6218	No Hepatic Impairment *n* = 4973	Hepatic Impairment *n* = 1245	*p*-Value
Age (years)	60 (50–68)	60 (50–68)	60 (50–67)	0.2
Male sex (%)	3187 (51%)	2595 (52%)	592 (48%)	0.003
Compounds				
Atezolizumab + bevacizumab	24 (0.4%)	1 (<0.1%)	23 (1.9%)	
Bevacizumab	131 (2.1%)	108 (2.2%)	23 (1.9%)	
Bevacizumab + capecitabine	28 (0.5%)	18 (0.4%)	10 (0.8%)	
Bevacizumab + capecitabine + oxaliplatin	72 (1.2%)	49 (1.0%)	23 (1.9%)	
Bevacizumab + fluorouracil + irinotecan	22 (0.4%)	13 (0.3%)	9 (0.7%)	
Bevacizumab + fluorouracil + irinotecan + oxaliplatin	38 (0.6%)	22 (0.4%)	16 (1.3%)	
Bevacizumab + paclitaxel	24 (0.4%)	6 (0.1%)	18 (1.5%)	
Bleomycine + cisplatin + etoposide	236 (3.8%)	190 (3.8%)	46 (3.7%)	
Bortezomib	116 (1.9%)	93 (1.9%)	23 (1.9%)	
Busulfan + fludarabine	152 (2.5%)	104 (2.1%)	48 (3.9%)	
Capecitabine + cisplatin + epirubicine	85 (1.4%)	78 (1.6%)	7 (0.6%)	
Capecitabine + oxaliplatin	174 (2.8%)	138 (2.8%)	36 (2.9%)	
Carboplatin	59 (1.0%)	44 (0.9%)	15 (1.2%)	
Carboplatin + etoposide	47 (0.8%)	35 (0.7%)	12 (1.0%)	
Carboplatin + gemcitabine	50 (0.8%)	44 (0.9%)	6 (0.5%)	
Carboplatin + paclitaxel	706 (11%)	597 (12%)	109 (8.8%)	
Carboplatin + pemetrexed	66 (1.1%)	56 (1.1%)	10 (0.8%)	
Carmustine + cytarabine + methotrexate + teniposide	20 (0.3%)	15 (0.3%)	5 (0.4%)	

Carmustine + methotrexate + teniposide	16 (0.3%)	9 (0.2%)	7 (0.6%)
cetuximab	125 (2.0%)	106 (2.1%)	19 (1.5%)
Cisplatin	990 (16%)	886 (18%)	104 (8.4%)
Cisplatin + cyclophosphamide + doxorubicin	11 (0.2%)	6 (0.1%)	5 (0.4%)
Cisplatin + etoposide	152 (2.5%)	120 (2.4%)	32 (2.6%)
Cisplatin + gemcitabine	131 (2.1%)	86 (1.7%)	45 (3.6%)
Cisplatin + paclitaxel	23 (0.4%)	18 (0.4%)	5 (0.4%)
Cisplatin + pemetrexed	75 (1.2%)	65 (1.3%)	10 (0.8%)
Cyclophosphamide	198 (3.2%)	160 (3.2%)	38 (3.1%)
Cyclophosphamide + docetaxel + epirubicine + fluorouracil	202 (3.3%)	177 (3.6%)	25 (2.0%)
Cyclophosphamide + doxorubicin	128 (2.1%)	101 (2.0%)	27 (2.2%)
Cyclophosphamide + doxorubicin + paclitaxel	59 (1.0%)	51 (1.0%)	8 (0.6%)
Cyclophosphamide + doxorubicin +rituximab + vincristine	182 (2.9%)	133 (2.7%)	49 (3.9%)
Cyclophosphamide + epirubicine + fluorouracil	32 (0.5%)	27 (0.5%)	5 (0.4%)
Cyclophosphamide + fludarabine	47 (0.8%)	35 (0.7%)	12 (1.0%)
Docetaxel	103 (1.7%)	82 (1.7%)	21 (1.7%)
Docetaxel + pertuzumab + trastuzumab	13 (0.2%)	6 (0.1%)	7 (0.6%)
Doxorubicin	38 (0.6%)	26 (0.5%)	12 (1.0%)
Doxorubicin liposomal	11 (0.2%)	4 (<0.1%)	7 (0.6%)
fludarabine	66 (1.1%)	44 (0.9%)	22 (1.8%)
Fluorouracil + irinotecan	17 (0.3%)	12 (0.2%)	5 (0.4%)
Fluorouracil + irinotecan + oxaliplatin	53 (0.9%)	34 (0.7%)	19 (1.5%)
Fluorouracil + oxaliplatin	19 (0.3%)	8 (0.2%)	11 (0.9%)
Gemcitabine	58 (0.9%)	35 (0.7%)	23 (1.9%)
Gemcitabine + paclitaxel + albumin nano	29 (0.5%)	19 (0.4%)	10 (0.8%)
Ipilimumab + nivolumab	262 (4.2%)	201 (4.0%)	61 (4.9%)
Irinotecan	15 (0.2%)	7 (0.1%)	8 (0.6%)

Melphalan	152 (2.5%)	139 (2.8%)	13 (1.0%)	
Methotrexate	76 (1.2%)	59 (1.2%)	17 (1.4%)	
Mitomycin	133 (2.1%)	121 (2.4%)	12 (1.0%)	
Nivolumab	159 (2.6%)	137 (2.8%)	22 (1.8%)	
Oxaliplatin	37 (0.6%)	30 (0.6%)	7 (0.6%)	
Paclitaxel	61 (1.0%)	34 (0.7%)	27 (2.2%)	
Panitumumab	26 (0.4%)	7 (0.1%)	19 (1.5%)	
Pembrolizumab	394 (6.4%)	330 (6.7%)	64 (5.2%)	
Rituximab	57 (0.9%)	39 (0.8%)	18 (1.5%)	
Vinorelbine	18 (0.3%)	8 (0.2%)	10 (0.8%)	
Lab values				
ALT (U/L)	22 (15–34)	19 (14–27)	43 (27–70)	<0.001
AST (U/L)	23 (18–31)	21 (17–25)	44 (36–62)	<0.001
Bilirubin (µmol/L)	8 (6–11)	8 (6–10)	9 (7–14)	<0.001
eGFR (ml/min)	88 (61–90)	87 (60–90)	90 (67–90)	0.003
gGT (U/L)	36 (22–70)	31 (20–53)	82 (38–203)	<0.001
Albumin (g/L)	39 (35–42)	40 (36–42)	38 (33–42)	<0.001
Leukocytes, ×10^9/L	8 (6–10)	8 (6–10)	8 (6–10)	0.083
Neutrophil count, ×10^9/L	5 (4–7)	5 (4–7)	5 (4–7)	0.013
Platelet count, ×10^9/L	275 (220–348)	275 (222–345)	273 (208–356)	0.13
Hb (mmol/L)	8 (7–9)	8 (8–9)	8 (7–9)	<0.001
**Follow-up events**				
Leukopenia	1623 (26%)	1259 (25%)	364 (29%)	0.005
Neutropenia	2090 (34%)	1609 (32%)	481 (39%)	<0.001
Thrombocytopenia	906 (15%)	683 (14%)	223 (18%)	<0.001
Anemia	714 (11%)	519 (10%)	195 (16%)	<0.001

Data presented as median (interquartile range) or *n* (%). ALT = alanine aminotransferase; AST = aspartate transaminase; eGFR = estimated glomerular filtration rate; gGT = gamma-glutamyl transferase; Hb = hemoglobulin.

Table S11. Baseline characteristics and follow-up events of the subset of patients included in renal impairment analyses that were treated with a compound from a pre-selected list of higher-risk compounds involved in renal clearance.

Baseline Characteristic	Overall *n* = 2929	No Renal Impairment *n* = 2714	Renal Impairment *n* = 215	*p*-Value
Age (years)	60 (51–67)	60 (51–67)	66 (57–71)	<0.001
Male sex (%)	1319 (45%)	1222 (45%)	97 (45%)	>0.9
ALT (U/L)	20 (15–30)	21 (15–30)	19 (13–28)	0.2
AST (U/L)	22 (18–29)	22 (18–29)	22 (17–30)	0.9
Bilirubin (µmol/L)	8 (6–10)	8 (6–10)	7 (6–10)	0.2
eGFR (ml/min)	87 (60–90)	89 (70–90)	49 (40–55)	<0.001
gGT (U/L)	34 (21–64)	34 (21–64)	32 (21–62)	0.9
Albumin (g/L)	39 (35–42)	39 (35–42)	39 (34–41)	0.003
Leukocytes, ×10^9/L	8 (6–10)	8 (6–10)	9 (7–11)	0.032
Neutrophil count, ×10^9/L	5 (4–7)	5 (4–7)	6 (4–8)	0.002
Platelet count, ×10^9/L	286 (230–356)	287 (230–356)	281 (229–360)	0.7
Hb (mmol/L)	8 (7–9)	8 (8–9)	7 (7–8)	<0.001
**Follow-up events**				
Leukopenia	751 (26%)	662 (24%)	89 (41%)	<0.001
Neutropenia	870 (30%)	778 (29%)	92 (43%)	<0.001

Baseline Characteristic	Overall<br>n = 2929	No Renal Impairment<br>n = 2714	Renal Impairment<br>n = 215	p-Value
Thrombocytopenia	407 (14%)	355 (13%)	52 (24%)	<0.001
Anemia	316 (11%)	269 (9.9%)	47 (22%)	<0.001

Data presented as median (interquartile range) or n (%). ALT = alanine aminotransferase; AST = aspartate transaminase; eGFR = estimated glomerular filtration rate; gGT = gamma-glutamyl transferase; Hb = hemoglobulin.

Table S12. Unadjusted and adjusted hazard ratios of impaired renal function categorized by severity in relation to hematological toxicity.

	Renal Impairment	Neutropenia	Thrombocytopenia	Anemia
Model 1	Mild	1.29 [1.00–1.66]	1.35 [0.97–1.86]	1.43 [1.01–2.03]
	Moderate	1.75 [1.25–2.45]	1.78 [1.23–2.59]	1.76 [1.11–2.79]
	Severe	1.45 [0.88–2.38]	1.24 [0.66–2.32]	2.69 [1.48–4.90]
Model 2	Mild	1.26 [0.98–1.64]	1.07 [0.77–1.48]	1.39 [0.97–1.98]
	Moderate	1.40 [0.99–1.98]	1.18 [0.80–1.75]	1.60 [1.00–2.57]
	Severe	1.03 [0.61–1.72]	0.62 [0.33–1.20]	1.95 [1.02–3.71]

Mild impairment of renal functioning was defined as a glomerular filtration rate (GFR) (CKD-EPI) of 45–59 mL/min, moderate impairment included a GFR of 30–44 mL/min, and severe impairment included a GFR of <30 mL/min. Hematological toxicities were defined as grade 3–4. Model 1 consisted of the unadjusted cox regression analysis. Model 2 consisted of the cox regression analysis adjusted for the chemotherapy protocol. Results are presented as hazard ratio (HR) with [95% CI]. Results are presented as hazard ratio (HR) with [95% CI].

Table S13. Baseline characteristics and follow-up events of the subset of patients included in hepatic impairment analyses that were treated with a compound from a pre-selected list of higher-risk compounds involved in hepatic metabolism.

Baseline Characteristic	Overall<br>n = 3462	No Hepatic Impairment<br>n = 2731	Hepatic Impairment<br>n = 731	p-Value
Age (years)	60 (49–68)	60 (49–68)	59 (50–67)	0.4
Male sex (%)	1674 (48%)	1366 (50%)	308 (42%)	<0.001
ALT (U/L)	21 (15–33)	19 (14–26)	41 (26–69)	<0.001
AST (U/L)	24 (19–31)	21 (17–25)	45 (36–65)	<0.001
Bilirubin (µmol/L)	8 (6–11)	8 (6–11)	9 (7–13)	<0.001
eGFR (ml/min)	86 (60–90)	85 (60–90)	89 (60–90)	0.001
gGT (U/L)	34 (21–67)	30 (19–50)	72 (35–183)	<0.001
Albumin (g/L)	39 (36–42)	40 (36–42)	38 (33–42)	<0.001
Leukocytes, ×10^9/L	8 (6–10)	8 (6–10)	8 (6–10)	0.006
Neutrophil count, ×10^9/L	5 (4–7)	5 (4–7)	5 (4–7)	0.005
Platelet count, ×10^9/L	275 (221–348)	274 (222–344)	282 (215–359)	0.13
Hb (mmol/L)	8 (7–9)	8 (8–9)	8 (7–9)	<0.001
**Follow-up events**				
Leukopenia	1230 (36%)	952 (35%)	278 (38%)	0.12
Neutropenia	1702 (49%)	1312 (48%)	390 (53%)	0.012
Thrombocytopenia	635 (18%)	485 (18%)	150 (21%)	0.10
Anemia	474 (14%)	344 (13%)	130 (18%)	<0.001

Data presented as median (interquartile range) or n (%). ALT = alanine aminotransferase; AST = aspartate transaminase; eGFR = estimated glomerular filtration rate; gGT = gamma-glutamyl transferase; Hb = hemoglobulin.

Table S14. Incidence of hematological toxicity during chemotherapy regimen compared between organ impairment subgroups.

Organ Impairment	Neutropenia	Thrombocytopenia	Anemia
Renal and hepatic impairment	28/85 (32.9%)	16/85 (18.8%)	20/85 (23.5%)
Renal impairment	138/398 (34.7%)	77/398 (19.3%)	70/398 (17.6%)
Hepatic impairment	422/1100 (38.4%)	174/1100 (15.8%)	150/1100 (13.6%)
No impairment	1476/4777 (30.9%)	597/4777 (12.5%)	425/4777(8.9%)

Supplementary S2

Overview of baseline characteristics and hematological toxicity events per chemotherapeutic regimen. Protocols are clustered at the level of unique combinations of administered compound(s).

Supplementary S2.1

Descriptive analytics of patients with renal impairment compared to patients with a normal renal function (≥60 mL/min eGFR).

Table S15 Baseline characteristics and follow-up events for patients with and without renal impairment receiving bortezomib + cyclophosphamide.

Baseline Characteristic	Overall n = 26	No Renal Impairment n = 14	Mild to Severe Renal Impairment n = 12	p-Value
Age (years)	65 (56–68)	65 (56–68)	65 (54–67)	0.7
Male sex (%)	21 (81%)	11 (79%)	10 (83%)	>0.9
ALT (U/L)	26 (20–36)	29 (24–38)	22 (16–33)	0.2
AST (U/L)	29 (21–34)	28 (27–32)	32 (18–38)	0.5
Bilirubin (µmol/L)	10 (5–18)	9 (5–11)	10 (4–25)	>0.9
eGFR (ml/min)	66 (39–89)	89 (75–90)	38 (31–52)	<0.001
gGT (U/L)	33 (22–62)	33 (30–41)	29 (15–76)	>0.9
Albumin (g/L)	37 (32–43)	36 (32–42)	37 (31–44)	0.6
Leukocytes, ×10^9/L	8 (5–11)	8 (5–9)	9 (6–12)	0.3
Neutrophil count, ×10^9/L	5 (3–8)	5 (3–8)	6 (3–9)	0.6
Platelet count, ×10^9/L	233 (195–300)	227 (155–292)	242 (197–306)	0.6
Hb (mmol/L)	8 (7–8)	8 (6–9)	8 (7–8)	0.8
**Follow-up events**				
Leukopenia	18 (69%)	10 (71%)	8 (67%)	>0.9
Neutropenia	18 (69%)	10 (71%)	8 (67%)	>0.9
Thrombocytopenia	18 (69%)	11 (79%)	7 (58%)	0.4
Anemia	9 (35%)	5 (36%)	4 (33%)	>0.9

Data presented as median (interquartile range) or n (%). ALT = alanine aminotransferase; AST = aspartate transaminase; eGFR = estimated glomerular filtration rate; gGT = gamma-glutamyl transferase; Hb = hemoglobulin.

Out of 26 patients treated with bortezomib + cyclophosphamide, 6 (23%) patients received a dosage of 1.3 mg/m^2 bortezomib and cyclophosphamide 300 mg/m^2 for an indication of AL-amyloidosis. Remaining patients were treated with 1.3 mg/m^2 bortezomib and cyclophosphamide 500 mg/m^2 for an indication of Multiple Myeloma.

Table S16 Baseline characteristics and follow-up events for patients with and without renal impairment receiving carboplatin + gemcitabine.

Baseline Characteristic	Overall $n = 50$	No renal Impairment $n = 39$	Mild to Severe Renal impairment $n = 11$	p-Value
Age (years)	69 (64–72)	69 (63–72)	68 (65–70)	0.8
Male sex (%)	30 (60%)	23 (59%)	7 (64%)	>0.9
ALT (U/L)	16 (13–25)	16 (12–25)	18 (14–20)	0.7
AST (U/L)	20 (16–24)	21 (16–24)	18 (16–44)	0.7
Bilirubin (µmol/L)	7 (6–10)	7 (7–10)	7 (6–10)	0.6
eGFR (ml/min)	73 (60–90)	82 (68–90)	51 (40–56)	<0.001
gGT (U/L)	38 (25–82)	35 (25–66)	55 (37–89)	0.2
Albumin (g/L)	38 (35–41)	38 (34–41)	39 (38–42)	0.4
Leukocytes, $\times 10^9$/L	9 (7–11)	10 (7–11)	8 (7–9)	0.2
Neutrophil count, $\times 10^9$/L	6 (4–8)	7 (4–9)	6 (4–6)	0.5
Platelet count, $\times 10^9$/L	325 (249–463)	335 (240–485)	303 (249–348)	0.4
Hb (mmol/L)	8 (7–9)	8 (7–9)	8 (7–9)	0.3
**Follow-up events**				
Leukopenia	19 (38%)	11 (28%)	8 (73%)	0.013
Neutropenia	29 (58%)	21 (54%)	8 (73%)	0.3
Thrombocytopenia	20 (40%)	16 (41%)	4 (36%)	>0.9
Anemia	20 (40%)	14 (36%)	6 (55%)	0.3

Data presented as median (interquartile range) or *n* (%). ALT = alanine aminotransferase; AST = aspartate transaminase; eGFR = estimated glomerular filtration rate; gGT = gamma-glutamyl transferase; Hb = hemoglobulin.

Out of a total of 50 patients, 43 (86%) were treated with carboplatin AUC = 5 + 1000 mg/m^2 gemcitabine for an indication of bladder cancer (*n* = 19) or non-small cell lung cancer (*n* = 24). Seven patients received carboplatin AUC = 4 + 1000 mg/m^2 gemcitabine for an indication of ovarium cancer.

Table S17 Baseline characteristics and follow-up events for patients with and without renal impairment receiving carboplatin + paclitaxel.

Baseline Characteristic	Overall $n = 706$	No Renal Impairment $n = 639$	Mild to Severe Renal Impairment $n = 67$	p-Value
Age (years)	66 (60–72)	66 (59–72)	69 (64–73)	0.006
Male sex (%)	253 (36%)	226 (35%)	27 (40%)	0.4
ALT (U/L)	19 (14–27)	19 (14–26)	18 (12–29)	0.3
AST (U/L)	22 (18–27)	23 (19–28)	21 (17–27)	0.13
Bilirubin (µmol/L)	8 (6–10)	8 (6–10)	8 (6–10)	0.6
eGFR (ml/min)	81 (60–90)	84 (66–90)	50 (43–55)	<0.001
gGT (U/L)	31 (20–56)	31 (20–57)	30 (21–48)	0.6
Albumin (g/L)	40 (36–42)	39 (37–42)	40 (35–41)	0.4
Leukocytes, $\times 10^9$/L	8 (7–10)	8 (7–10)	9 (6–11)	0.051
Neutrophil count, $\times 10^9$/L	5 (4–7)	5 (4–7)	6 (4–8)	0.016
Platelet count, $\times 10^9$/L	288 (234–352)	288 (232–352)	292 (248–368)	0.4
Hb (mmol/L)	8 (8–9)	8 (8–9)	8 (6–9)	<0.001
**Follow-up events**				
Leukopenia	219 (31%)	196 (31%)	23 (34%)	0.6
Neutropenia	256 (36%)	232 (36%)	24 (36%)	>0.9
Thrombocytopenia	71 (10%)	64 (10%)	7 (10%)	>0.9
Anemia	69 (9.8%)	62 (9.7%)	7 (10%)	>0.9

Data presented as median (interquartile range) or *n* (%). ALT = alanine aminotransferase; AST = aspartate transaminase; eGFR = estimated glomerular filtration rate; gGT = gamma-glutamyl transferase; Hb = hemoglobulin.

Out of a total of 706 patients receiving a carboplatin + paclitaxel protocol, 406 (58%) patients received carboplatin AUC = 2 + 50 mg/m² paclitaxel for an indication of esophageal cancer. In total 291 patients were treated with this compound combination for an indication of ovarium cancer, 233 patients with carboplatin AUC = 6 + 175 mg/m² paclitaxel and 58 patients with carboplatin AUC = 5 + 175 mg/m². Details on remaining protocols are not provided due to a large diversification of protocols and patient pseudonymization.

Table S18 Baseline characteristics and follow-up events for patients with and without renal impairment receiving cyclophosphamide.

Baseline Characteristic	Overall *n* = 198	No Renal Impairment *n* = 171	Mild to Severe Renal impairment *n* = 27	*p*-Value
Age (years)	51 (38–60)	49 (38–59)	52 (42–65)	0.13
Male sex (%)	98 (49%)	83 (49%)	15 (56%)	0.5
ALT (U/L)	26 (17–38)	26 (17–38)	29 (18–38)	0.5
AST (U/L)	22 (16–31)	22 (16–30)	24 (18–37)	0.4
Bilirubin (μmol/L)	7 (6–9)	8 (6–10)	7 (6–8)	0.5
eGFR (ml/min)	90 (60–90)	90 (84–90)	45 (30–55)	<0.001
gGT (U/L)	32 (21–61)	32 (20–65)	35 (24–47)	0.5
Albumin (g/L)	35 (28–40)	36 (28–40)	35 (22–39)	0.2
Leukocytes, ×10⁹/L	9 (6–13)	8 (6–12)	10 (6–14)	0.3
Neutrophil count, ×10⁹/L	6 (4–10)	6 (4–10)	8 (5–11)	0.2
Platelet count, ×10⁹/L	279 (224–354)	283 (224–356)	257 (205–308)	0.3
Hb (mmol/L)	8 (7–9)	8 (7–9)	7 (6–8)	0.006
**Follow-up events**				
Leukopenia	74 (37%)	64 (37%)	10 (37%)	>0.9
Neutropenia	77 (39%)	66 (39%)	11 (41%)	>0.9
Thrombocytopenia	71 (36%)	61 (36%)	10 (37%)	>0.9
Anemia	35 (18%)	30 (18%)	5 (19%)	>0.9

Data presented as median (interquartile range) or *n* (%). ALT = alanine aminotransferase; AST = aspartate transaminase; eGFR = estimated glomerular filtration rate; gGT = gamma-glutamyl transferase; Hb = hemoglobulin.

Out of 198 patients treated with a cyclophosphamide monotherapy, 75 patients received 750 mg/m² cyclophosphamide for various indications. Sixty-six patients received 2000 mg/m² cyclophosphamide for stem cell mobilization in multiple myeloma. Twenty-nine patients received 500 mg/m² for an indication of proliferative lupus nephritis. Details on remaining protocols are not provided due to a large diversification of protocols and patient pseudonymization.

Table S19 Baseline characteristics and follow-up events for patients with and without renal impairment receiving cyclophosphamide + doxorubicin.

Baseline Characteristic	Overall *n* = 128	No Renal Impairment *n* = 120	Mild to Severe Renal Impairment *n* = 8	*p*-Value
Age (years)	52 (47–59)	51 (46–58)	66 (60–66)	0.007
Male sex (%)	9 (7.0%)	6 (5.0%)	3 (38%)	0.011

Baseline Characteristic	Overall $n = 128$	No Renal Impairment $n = 120$	Mild to Severe Renal Impairment $n = 8$	p-Value
ALT (U/L)	21 (15–30)	21 (15–30)	22 (14–29)	>0.9
AST (U/L)	24 (18–31)	23 (18–30)	28 (24–64)	0.077
Bilirubin (μmol/L)	7 (6–10)	7 (6–10)	6 (6–9)	0.8
eGFR (ml/min)	80 (60–90)	84 (60–90)	55 (45–57)	<0.001
gGT (U/L)	30 (17–58)	31 (17–58)	23 (19–44)	0.6
Albumin (g/L)	40 (36–43)	41 (37–44)	38 (35–39)	0.2
Leukocytes, $\times 10^9$/L	7 (6–9)	7 (6–9)	8 (7–13)	0.2
Neutrophil count, $\times 10^9$/L	5 (4–6)	5 (4–6)	7 (5–9)	0.050
Platelet count, $\times 10^9$/L	286 (221–340)	292 (225–342)	221 (151–259)	0.035
Hb (mmol/L)	8 (8–9)	8 (8–9)	8 (7–9)	0.2
**Follow-up events**				
Leukopenia	64 (50%)	57 (48%)	7 (88%)	0.062
Neutropenia	80 (63%)	74 (62%)	6 (75%)	0.7
Thrombocytopenia	23 (18%)	20 (17%)	3 (38%)	0.2
Anemia	18 (14%)	13 (11%)	5 (63%)	0.001

Data presented as median (interquartile range) or n (%). ALT = alanine aminotransferase; AST = aspartate transaminase; eGFR = estimated glomerular filtration rate; gGT = gamma-glutamyl transferase; Hb = hemoglobulin.

Almost all patients (117/128, 91%) receiving the combination therapy of cyclophosphamide + doxorubicin, were treated for an indication of mamma carcinoma. Dosing schedules included 600 mg/m^2 cyclophosphamide + 60 mg/m^2 doxorubicin administered 1x a week for 2 to 3 weeks. Details on remaining protocols are not provided due to a large diversification of protocols and patient pseudonymization.

Table S20 Baseline characteristics and follow-up events for patients with and without renal impairment receiving cyclophosphamide + doxorubicin + rituximab + vincristine.

Baseline Characteristic	Overall $n = 182$	No Renal Impairment $n = 160$	Mild to Severe Renal Impairment $n = 22$	p-Value
Age (years)	61 (49–70)	61 (49–70)	61 (43–72)	0.9
Male sex (%)	119 (65%)	112 (70%)	7 (32%)	<0.001
ALT (U/L)	21 (15–37)	22 (16–37)	15 (12–22)	0.007
AST (U/L)	24 (18–41)	24 (19–41)	21 (16–50)	0.6
Bilirubin (μmol/L)	8 (6–11)	9 (6–11)	6 (5–11)	0.2
eGFR (ml/min)	83 (60–90)	85 (69–90)	47 (32–53)	<0.001
gGT (U/L)	33 (21–61)	35 (21–61)	26 (16–58)	0.2
Albumin (g/L)	38 (34–42)	38 (34–42)	38 (27–41)	0.6
Leukocytes, $\times 10^9$/L	9 (7–12)	9 (7–12)	9 (6–11)	>0.9
Neutrophil count, $\times 10^9$/L	6 (4–8)	5 (4–8)	7 (5–9)	0.3
Platelet count, $\times 10^9$/L	283 (221–375)	286 (223–375)	271 (217–406)	>0.9
Hb (mmol/L)	8 (7–9)	8 (7–9)	7 (7–7)	<0.001
**Follow-up events**				
Leukopenia	99 (54%)	81 (51%)	18 (82%)	0.012
Neutropenia	128 (70%)	109 (68%)	19 (86%)	0.13
Thrombocytopenia	47 (26%)	40 (25%)	7 (32%)	0.7

Anemia	38 (21%)	29 (18%)	9 (41%)	0.023

Data presented as median (interquartile range) or *n* (%). ALT = alanine aminotransferase; AST = aspartate transaminase; eGFR = estimated glomerular filtration rate; gGT = gamma-glutamyl transferase; Hb = hemoglobulin.

All patients receiving this compound combination were treated with 750 mg/m^2 cyclophosphamide, 50 mg/m^2 doxorubicin, 375 mg/m^2 rituximab, and 2 mg/m^2 vincristine. This protocol was used for the indication of mantle cell lymphoma and diffuse large B-cell lymphoma.

Table S21 Baseline characteristics and follow-up events for patients with and without renal impairment receiving melphalan.

**Baseline Characteristic**	**Overall** *n* **= 152**	**No Renal Impairment** *n* **= 136**	**Mild to Severe Renal Impairment** *n* **= 16**	***p*-Value**
Age (years)	60 (56–64)	60 (56–64)	62 (55–63)	>0.9
Male sex (%)	107 (70%)	99 (73%)	8 (50%)	0.081
ALT (U/L)	18 (13–24)	18 (13–24)	17 (12–26)	0.7
AST (U/L)	19 (14–24)	19 (14–24)	19 (16–24)	0.8
Bilirubin (μmol/L)	7 (6–10)	8 (6–10)	7 (5–9)	0.3
eGFR (ml/min)	88 (60–90)	90 (70–90)	46 (41–49)	<0.001
gGT (U/L)	32 (21–47)	32 (21–46)	38 (27–53)	0.5
Albumin (g/L)	40 (37–42)	40 (37–43)	39 (31–40)	0.3
Leukocytes, ×10^9/L	6 (5–7)	6 (4–7)	6 (5–7)	0.4
Neutrophil count, ×10^9/L	4 (3–5)	4 (3–5)	4 (3–5)	0.5
Platelet count, ×10^9/L	249 (205–321)	256 (206–316)	239 (203–410)	0.7
Hb (mmol/L)	8 (7–9)	8 (7–9)	7 (7–8)	<0.001
**Follow-up events**				
Leukopenia	51 (34%)	44 (32%)	7 (44%)	0.5
Neutropenia	53 (35%)	46 (34%)	7 (44%)	0.6
Thrombocytopenia	51 (34%)	44 (32%)	7 (44%)	0.5
Anemia	14 (9.2%)	12 (8.8%)	2 (13%)	0.6

Data presented as median (interquartile range) or *n* (%). ALT = alanine aminotransferase; AST = aspartate transaminase; eGFR = estimated glomerular filtration rate; gGT = gamma-glutamyl transferase; Hb = hemoglobulin.

Out of 152 patients, 141 patients were treated with 100 mg/m^2 for an indication of autologous stem cell transplantation in multiple myeloma patients. Remaining patients were treated with 70 mg/m^2 for various indications.

Table S22 Baseline characteristics and follow-up events for patients with and without renal impairment receiving mitomycin.

**Baseline Characteristic**	**Overall** *n* **= 133**	**No Renal Impairment** *n* **= 121**	**Mild to Severe Renal Impairment** *n* **= 12**	***p*-Value**
Age (years)	63 (55–69)	63 (55–68)	69 (57–75)	0.2
Male sex (%)	52 (39%)	45 (37%)	7 (58%)	0.2
ALT (U/L)	21 (14–28)	20 (14–28)	21 (16–22)	>0.9
AST (U/L)	23 (20–28)	23 (20–28)	23 (23–28)	0.6
Bilirubin (μmol/L)	7 (5–11)	7 (5–11)	7 (6–13)	0.6
eGFR (ml/min)	86 (78–90)	89 (80–90)	52 (41–57)	<0.001
gGT (U/L)	33 (20–69)	33 (20–69)	39 (25–47)	>0.9
Albumin (g/L)	33 (27–42)	33 (27–41)	34 (28–44)	0.7

Leukocytes, ×10⁹/L	8 (7–10)	8 (7–10)	8 (7–9)	0.8
Neutrophil count, ×10⁹/L	5 (4–7)	5 (4–7)	5 (4–6)	0.5
Platelet count, ×10⁹/L	277 (231–348)	276 (231–348)	285 (237–378)	0.7
Hb (mmol/L)	8 (7–9)	8 (7–9)	7 (6–8)	>0.9
**Follow-up events**				
Leukopenia	8 (6.0%)	6 (5.0%)	2 (17%)	0.2
Neutropenia	12 (9.0%)	10 (8.3%)	2 (17%)	0.3
Thrombocytopenia	4 (3.0%)	3 (2.5%)	1 (8.3%)	0.3
Anemia	30 (23%)	25 (21%)	5 (42%)	0.14

Data presented as median (interquartile range) or *n* (%). ALT = alanine aminotransferase; AST = aspartate transaminase; eGFR = estimated glomerular filtration rate; gGT = gamma-glutamyl transferase; Hb = hemoglobulin.

Out of 133 patients, 110 patients received in total 35 mg/m² for an indication of colon cancer. Twenty-two patients received a one-time dose of 12 mg/m² for an indication of anal cancer. Details on remaining protocols are not provided due to a large diversification of protocols and patient pseudonymization.

Supplementary S2.2

Descriptive analytics of patients with hepatic impairment compared to patients with a normal hepatic function.

Table S23 Baseline characteristics and follow-up events for patients with and without hepatic impairment receiving bevacizumab + fluorouracil + irinotecan + oxaliplatin.

Baseline Characteristic	Overall *n* = 38	No Hepatic Impairment *n* = 22	Hepatic Impairment *n* = 16	*p*-Value
Age (years)	54 (45–61)	53 (39–59)	57 (52–62)	0.095
Male sex (%)	16 (42%)	10 (45%)	6 (38%)	0.6
ALT (U/L)	31 (18–51)	21 (14–30)	51 (41–58)	<0.001
AST (U/L)	30 (23–54)	23 (19–25)	67 (43–75)	<0.001
Bilirubin (μmol/L)	8 (6–11)	8 (5–11)	9 (7–10)	>0.9
eGFR (ml/min)				0.4
gGT (U/L)	71 (32–172)	43 (25–64)	201 (130–498)	<0.001
Albumin (g/L)	39 (34–41)	40 (36–42)	36 (33–41)	0.4
Leukocytes, ×10⁹/L	8 (7–11)	8 (7–9)	9 (8–13)	0.11
Neutrophil count, ×10⁹/L	6 (4–8)	6 (4–7)	7 (5–8)	0.13
Platelet count, ×10⁹/L	342 (263–413)	328 (246–382)	390 (292–507)	0.2
Hb (mmol/L)	8 (7–9)	8 (7–9)	8 (7–9)	0.5
**Follow-up events**				
Leukopenia	5 (13%)	3 (14%)	2 (13%)	>0.9
Neutropenia	18 (47%)	9 (41%)	9 (56%)	0.5
Thrombocytopenia	1 (2.6%)	1 (4.5%)	0 (0%)	>0.9
Anemia	4 (11%)	3 (14%)	1 (6.3%)	0.6

Data presented as median (interquartile range) or *n* (%). ALT = alanine aminotransferase; AST = aspartate transaminase; eGFR = estimated glomerular filtration rate; gGT = gamma-glutamyl transferase; Hb = hemoglobulin.

All patients were treated for an indication of colorectal cancer with 5 mg/m² bevacizumab + 3200 mg/m² fluorouracil + 165 mg/m² irinotecan + 85 mg/m² oxaliplatin.

Table S24 Baseline characteristics and follow-up events for patients with and without hepatic impairment receiving bleomycin + cisplatin + etoposide.

Baseline Characteristic	Overall<br>*n* = 236	No Hepatic Impairment<br>*n* = 190	Hepatic Impairment<br>*n* = 46	*p*-Value
Age (years)	32 (27–37)	31 (26–37)	33 (28–38)	0.11
Male sex (%)	230 (97%)	186 (98%)	44 (96%)	0.3
ALT (U/L)	24 (16–39)	22 (16–33)	46 (28–78)	<0.001
AST (U/L)	25 (21–32)	23 (20–27)	42 (36–63)	<0.001
Bilirubin (µmol/L)	9 (7–11)	10 (8–11)	9 (7–11)	0.7
eGFR (ml/min)	90 (88–90)	90 (85–90)	90 (90–90)	0.080
gGT (U/L)	26 (19–45)	24 (17–35)	47 (24–75)	<0.001
Albumin (g/L)	44 (41–46)	43 (41–45)	44 (39–46)	0.6
Leukocytes, $\times 10^9$/L	7 (6–9)	7 (6–9)	7 (6–9)	0.6
Neutrophil count, $\times 10^9$/L	4 (3–6)	4 (3–6)	5 (3–6)	0.14
Platelet count, $\times 10^9$/L	263 (218–325)	263 (215–324)	264 (234–381)	0.3
Hb (mmol/L)	9 (9–10)	9 (9–10)	9 (8–10)	0.033
**Follow-up events**				
Leukopenia	116 (49%)	90 (47%)	26 (57%)	0.3
Neutropenia	210 (89%)	168 (88%)	42 (91%)	0.8
Thrombocytopenia	27 (11%)	17 (8.9%)	10 (22%)	0.029
Anemia	16 (6.8%)	9 (4.7%)	7 (15%)	0.019

Data presented as median (interquartile range) or *n* (%). ALT = alanine aminotransferase; AST = aspartate transaminase; eGFR = estimated glomerular filtration rate; gGT = gamma-glutamyl transferase; Hb = hemoglobulin.

All patients were treated with a combination therapy of 30,000 IU bleomycin (day 2, 8 and 15) + 20 mg/m^2 cisplatin (day 1–5) + 100 mg/m^2 etoposide (day 1–5) for the indication of testicular cancer.

Table S25 Baseline characteristics and follow-up events for patients with and without hepatic impairment receiving bortezomib.

Baseline Characteristic	Overall<br>*n* = 116	No Hepatic Impairment<br>*n* = 93	Hepatic Impairment<br>*n* = 23	*p*-Value
Age (years)	62 (54–69)	62 (55–69)	62 (53–68)	0.5
Male sex (%)	68 (59%)	57 (61%)	11 (48%)	0.2
ALT (U/L)	22 (15–32)	20 (14–29)	37 (26–74)	<0.001
AST (U/L)	24 (19–34)	20 (17–24)	40 (34–72)	<0.001
Bilirubin (µmol/L)	9 (6–14)	8 (6–12)	10 (6–23)	0.2
eGFR (ml/min)	60 (41–85)	60 (41–84)	60 (36–90)	>0.9
gGT (U/L)	33 (22–61)	29 (19–48)	51 (24–159)	0.014
Albumin (g/L)	35 (30–39)	36 (31–39)	33 (28–39)	0.3
Leukocytes, $\times 10^9$/L	7 (5–9)	6 (5–9)	8 (5–11)	0.10
Neutrophil count, $\times 10^9$/L	4 (3–6)	4 (3–6)	6 (3–9)	0.063
Platelet count, $\times 10^9$/L	223 (161–296)	222 (164–303)	228 (148–274)	0.5
Hb (mmol/L)	7 (6–8)	7 (6–8)	7 (5–8)	0.2
**Follow-up events**				
Leukopenia	56 (48%)	45 (48%)	11 (48%)	>0.9
Neutropenia	59 (51%)	47 (51%)	12 (52%)	>0.9
Thrombocytopenia	65 (56%)	53 (57%)	12 (52%)	0.9

Baseline Characteristic	Overall $n = 116$	No Hepatic Impairment $n = 93$	Hepatic Impairment $n = 23$	p-Value
Anemia	31 (27%)	23 (25%)	8 (35%)	0.5

Data presented as median (interquartile range) or n (%). ALT = alanine aminotransferase; AST = aspartate transaminase; eGFR = estimated glomerular filtration rate; gGT = gamma-glutamyl transferase; Hb = hemoglobulin.

The majority of patients (n = 104) treated with bortezomib monotherapy for the indication of Multiple Myeloma received a dose of 1.3 mg/m^2, while remaining patients received a dose of 1.6 mg/m^2.

Table S26 Baseline characteristics and follow-up events for patients with and without hepatic impairment receiving capecitabine + cisplatin + epirubicin.

Baseline Characteristic	Overall $n = 85$	No Hepatic Impairment $n = 78$	Hepatic Impairment $n = 7$	p-Value
Age (years)	60 (53–67)	60 (52–67)	63 (55–69)	0.5
Male sex (%)	58 (68%)	56 (72%)	2 (29%)	0.031
ALT (U/L)	20 (15–30)	19 (14–24)	32 (29–60)	0.002
AST (U/L)	19 (15–26)	18 (14–24)	32 (21–52)	0.002
Bilirubin (µmol/L)	9 (7–12)	9 (6–11)	16 (12–23)	0.015
eGFR (ml/min)	60 (60–74)	60 (60–74)	60 (60–89)	0.9
gGT (U/L)	28 (20–50)	26 (19–49)	62 (25–89)	0.080
Albumin (g/L)	40 (38–42)	40 (38–42)	43 (38–44)	0.3
Leukocytes, ×10^9/L	8 (6–10)	8 (6–10)	6 (5–8)	0.036
Neutrophil count, ×10^9/L	5 (4–6)	5 (4–6)	4 (3–5)	0.089
Platelet count, ×10^9/L	269 (235–351)	270 (242–351)	243 (143–383)	0.3
Hb (mmol/L)	9 (8–9)	9 (8–9)	9 (8–10)	0.2
**Follow-up events**				
Leukopenia	14 (16%)	10 (13%)	4 (57%)	0.013
Neutropenia	35 (41%)	30 (38%)	5 (71%)	0.12
Thrombocytopenia	4 (4.7%)	3 (3.8%)	1 (14%)	0.3
Anemia	3 (3.5%)	2 (2.6%)	1 (14%)	0.2

Data presented as median (interquartile range) or n (%). ALT = alanine aminotransferase; AST = aspartate transaminase; eGFR = estimated glomerular filtration rate; gGT = gamma-glutamyl transferase; Hb = hemoglobulin.

The majority of patients (n = 78) treated with this combination therapy received a dosage of 625 mg/m^2 capecitabine + 60 mg/m^2 cisplatin + 50 mg/m^2 epirubicin. Remaining patients received a dosage of 1000 mg/m^2 capecitabine + 60 mg/m^2 cisplatin + 50 mg/m^2 epirubicin. All patients were treated for an indication of esophageal or stomach cancer.

Table S27 Baseline characteristics and follow-up events for patients with and without hepatic impairment receiving carboplatin + etoposide.

Baseline Characteristic	Overall $n = 47$	No Hepatic Impairment $n = 35$	Hepatic Impairment $n = 12$	p-Value
Age (years)	67 (60–75)	68 (60–74)	66 (60–77)	0.8
Male sex (%)	28 (60%)	22 (63%)	6 (50%)	0.5
ALT (U/L)	20 (14–36)	16 (13–23)	39 (24–45)	0.003
AST (U/L)	27 (21–32)	24 (19–28)	40 (32–58)	<0.001
Bilirubin (µmol/L)	8 (6–11)	8 (6–10)	11 (8–13)	0.015

eGFR (ml/min)	76 (62–87)	76 (62–87)	72 (64–88)	>0.9
gGT (U/L)	35 (22–53)	31 (22–48)	47 (26–141)	0.088
Albumin (g/L)	39 (38–41)	39 (38–41)	38 (37–42)	0.4
Leukocytes, ×10^9/L	9 (7–11)	9 (7–11)	8 (7–11)	0.9
Neutrophil count, ×10^9/L	6 (4–9)	6 (4–9)	6 (5–9)	0.5
Platelet count, ×10^9/L	282 (218–351)	289 (234–351)	233 (188–285)	0.13
Hb (mmol/L)	8 (8–9)	8 (8–9)	9 (7–9)	0.9
**Follow-up events**				
Leukopenia	20 (43%)	14 (40%)	6 (50%)	0.8
Neutropenia	34 (72%)	24 (69%)	10 (83%)	0.5
Thrombocytopenia	24 (51%)	16 (46%)	8 (67%)	0.4
Anemia	13 (28%)	8 (23%)	5 (42%)	0.3

Data presented as median (interquartile range) or *n* (%). ALT = alanine aminotransferase; AST = aspartate transaminase; eGFR = estimated glomerular filtration rate; gGT = gamma-glutamyl transferase; Hb = hemoglobulin.

The majority of patients (*n* = 38) were treated for an indication of small cell lung cancer with carboplatin (AUC = 5) and 120 mg/m^2 etoposide. Several patients (*n* = 6) with an indication of testicular cancer were also treated with the same dosage. Details on remaining protocols are not provided due to a large diversification of protocols and patient pseudonymization.

Table S28 Baseline characteristics and follow-up events for patients with and without hepatic impairment receiving carboplatin + paclitaxel.

**Baseline Characteristic**	**Overall** *n* **= 706**	**No Hepatic Impairment** *n* **= 597**	**Hepatic Impairment** *n* **= 109**	***p*-Value**
Age (years)	66 (60–72)	66 (60–72)	66 (58–71)	0.5
Male sex (%)	253 (36%)	228 (38%)	25 (23%)	0.002
ALT (U/L)	19 (14–27)	17 (13–23)	33 (21–64)	<0.001
AST (U/L)	22 (18–27)	21 (17–25)	38 (33–48)	<0.001
Bilirubin (µmol/L)	8 (6–10)	8 (6–10)	8 (6–10)	0.7
eGFR (ml/min)	81 (60–90)	80 (60–90)	84 (60–90)	0.4
gGT (U/L)	31 (20–56)	29 (19–50)	52 (29–109)	<0.001
Albumin (g/L)	40 (36–42)	40 (37–42)	39 (35–42)	0.5
Leukocytes, ×10^9/L	8 (7–10)	8 (6–10)	8 (7–10)	0.2
Neutrophil count, ×10^9/L	5 (4–7)	5 (4–7)	6 (4–7)	0.2
Platelet count, ×10^9/L	288 (234–352)	286 (231–348)	298 (251–373)	0.074
Hb (mmol/L)	8 (8–9)	8 (8–9)	8 (8–9)	0.013
**Follow-up events**				
Leukopenia	219 (31%)	176 (29%)	43 (39%)	0.050
Neutropenia	256 (36%)	210 (35%)	46 (42%)	0.2
Thrombocytopenia	71 (10%)	60 (10%)	11 (10%)	>0.9
Anemia	69 (9.8%)	51 (8.5%)	18 (17%)	0.016

Data presented as median (interquartile range) or *n* (%). ALT = alanine aminotransferase; AST = aspartate transaminase; eGFR = estimated glomerular filtration rate; gGT = gamma-glutamyl transferase; Hb = hemoglobulin.

The majority of patients (*n* = 406) treated with this compound combination were treated for an indication of esophageal cancer with carboplatin (AUC = 2) and 50 mg/m^2 paclitaxel 5 or 6 times a week. Most patients (*n* = 233) with an indication of ovarium cancer were treated with carboplatin (AUC = 6) and 175 mg/m^2 paclitaxel. Fifty-eight patients with an indication of ovarium cancer were treated with

carboplatin (AUC = 5) and 175 mg/m^2 paclitaxel. Details on remaining protocols are not provided due to a large diversification of protocols and patient pseudonymization.

Table S29 Baseline characteristics and follow-up events for patients with and without hepatic impairment receiving carboplatin + pemetrexed.

Baseline Characteristic	Overall *n* = 66	No Hepatic Impairment *n* = 56	Hepatic Impairment *n* = 10	*p*-Value
Age (years)	65 (58–70)	65 (57–71)	64 (59–70)	0.9
Male sex (%)	33 (50%)	30 (54%)	3 (30%)	0.2
ALT (U/L)	21 (17–31)	20 (16–25)	65 (51–90)	0.001
AST (U/L)	22 (18–27)	21 (17–24)	53 (45–69)	<0.001
Bilirubin (µmol/L)	8 (6–11)	8 (6–12)	8 (3–10)	0.4
eGFR (ml/min)	83 (60–90)	83 (60–90)	84 (60–90)	0.8
gGT (U/L)	50 (26–98)	47 (25–78)	103 (82–213)	0.004
Albumin (g/L)	39 (36–42)	40 (37–42)	38 (34–41)	0.6
Leukocytes, ×10^9/L	11 (8–14)	11 (8–14)	11 (7–17)	0.9
Neutrophil count, ×10^9/L	8 (5–11)	8 (5–10)	9 (5–15)	0.8
Platelet count, ×10^9/L	340 (263–394)	342 (264–396)	296 (257–372)	0.5
Hb (mmol/L)	8 (8–9)	8 (8–9)	8 (7–8)	0.2
**Follow-up events**				
Leukopenia	17 (26%)	13 (23%)	4 (40%)	0.3
Neutropenia	26 (39%)	21 (38%)	5 (50%)	0.5
Thrombocytopenia	16 (24%)	13 (23%)	3 (30%)	0.7
Anemia	11 (17%)	10 (18%)	1 (10%)	>0.9

Data presented as median (interquartile range) or *n* (%). ALT = alanine aminotransferase; AST = aspartate transaminase; eGFR = estimated glomerular filtration rate; gGT = gamma-glutamyl transferase; Hb = hemoglobulin.

All patients received a regimen consisting of carboplatin (AUC = 5) and 500 mg/m^2 pemetrexed for an indication of non-small cell lung cancer.

Table S30 Baseline characteristics and follow-up events for patients with and without hepatic impairment receiving carmustine + methotrexate + teniposide.

Baseline Characteristic	Overall *n* = 16	No Hepatic Impairment *n* = 9	Hepatic Impairment *n* = 7	*p*-Value
Age (years)	64 (51–66)	66 (60–67)	52 (42–64)	0.088
Male sex (%)	8 (50%)	5 (56%)	3 (43%)	>0.9
ALT (U/L)	71 (28–145)	33 (21–58)	144 (71–152)	0.012
AST (U/L)	29 (21–45)	23 (17–24)	51 (38–84)	0.001
Bilirubin (µmol/L)	10 (7–14)	9 (7–10)	15 (12–28)	0.051
eGFR (ml/min)				0.064
gGT (U/L)	57 (33–73)	40 (28–62)	67 (52–169)	0.039
Albumin (g/L)	37 (34–40)	38 (37–40)	35 (30–37)	0.088
Leukocytes, ×10^9/L	11 (8–15)	10 (8–16)	11 (9–15)	>0.9
Neutrophil count, ×10^9/L	8 (6–13)	8 (7–11)	8 (6–13)	>0.9
Platelet count, ×10^9/L	237 (190–289)	238 (212–299)	237 (148–278)	0.5
Hb (mmol/L)	9 (8–9)	8 (7–9)	9 (9–10)	0.023
**Follow-up events**				
Leukopenia	15 (94%)	8 (89%)	7 (100%)	>0.9

Neutropenia	15 (94%)	8 (89%)	7 (100%)	>0.9
Thrombocytopenia	8 (50%)	4 (44%)	4 (57%)	>0.9
Anemia	5 (31%)	2 (22%)	3 (43%)	0.6

Data presented as median (interquartile range) or *n* (%). ALT = alanine aminotransferase; AST = aspartate transaminase; eGFR = estimated glomerular filtration rate; gGT = gamma-glutamyl transferase; Hb = hemoglobulin.

All patients received a regimen consisting of 100 mg/m^2 carmustine, 3000 mg/m^2 methotrexate, and 100 mg/m^2 teniposide for an indication of non-Hodgkin lymphoma.

Table S31 Baseline characteristics and follow-up events for patients with and without hepatic impairment receiving cisplatin + etoposide.

**Baseline Characteristic**	**Overall** *n* **= 152**	**No Hepatic Impairment** *n* **= 120**	**Hepatic Impairment** *n* **= 32**	***p*-Value**
Age (years)	56 (43–66)	56 (41–66)	57 (49–65)	>0.9
Male sex (%)	110 (72%)	89 (74%)	21 (66%)	0.3
ALT (U/L)	23 (16–34)	21 (15–28)	45 (26–58)	<0.001
AST (U/L)	24 (19–31)	21 (17–26)	42 (38–57)	<0.001
Bilirubin (µmol/L)	9 (6–12)	9 (6–11)	10 (7–15)	0.089
eGFR (ml/min)	90 (77–90)	90 (72–90)	90 (84–90)	0.3
gGT (U/L)	36 (22–63)	31 (20–44)	67 (48–152)	<0.001
Albumin (g/L)	42 (37–44)	41 (37–44)	43 (37–45)	0.4
Leukocytes, $\times 10^9$/L	8 (6–10)	8 (6–9)	9 (6–12)	0.2
Neutrophil count, $\times 10^9$/L	5 (4–7)	5 (4–6)	6 (4–8)	0.5
Platelet count, $\times 10^9$/L	290 (236–357)	286 (235–356)	302 (237–369)	0.9
Hb (mmol/L)	9 (8–9)	9 (8–9)	9 (8–10)	0.7
**Follow-up events**				
Leukopenia	82 (54%)	65 (54%)	17 (53%)	>0.9
Neutropenia	135 (89%)	106 (88%)	29 (91%)	>0.9
Thrombocytopenia	34 (22%)	28 (23%)	6 (19%)	0.8
Anemia	28 (18%)	27 (23%)	1 (3.1%)	0.024

Data presented as median (interquartile range) or *n* (%). ALT = alanine aminotransferase; AST = aspartate transaminase; eGFR = estimated glomerular filtration rate; gGT = gamma-glutamyl transferase; Hb = hemoglobulin.

Eighty-three patients receiving a regimen of 60 mg/m^2 cisplatin and 120 mg/m^2 etoposide were treated for an indication of small-cell carcinoma or neuroendocrine cancer. Sixty-one patients received a regimen of 20 mg/m^2 cisplatin and 100 mg/m^2 etoposide for an indication of testicular cancer. Details on remaining protocols are not provided due to a large diversification of protocols and patient pseudonymization.

Table S32 Baseline characteristics and follow-up events for patients with and without hepatic impairment receiving cisplatin + gemcitabine.

**Baseline Characteristic**	**Overall** *n* **= 131**	**No Hepatic Impairment** *n* **= 86**	**Hepatic Impairment** *n* **= 45**	***p*-Value**
Age (years)	62 (53–67)	61 (53–67)	62 (53–69)	0.8
Male sex (%)	74 (56%)	55 (64%)	19 (42%)	0.017
ALT (U/L)	25 (17–36)	20 (15–27)	41 (28–98)	<0.001
AST (U/L)	28 (19–40)	22 (17–27)	57 (38–92)	<0.001
Bilirubin (µmol/L)	9 (7–12)	8 (6–11)	11 (7–16)	0.002

eGFR (ml/min)	89 (69–90)	89 (64–90)	89 (80–90)	0.5
gGT (U/L)	77 (33–236)	46 (26–90)	290 (125–560)	<0.001
Albumin (g/L)	39 (34–42)	39 (35–42)	37 (30–42)	0.3
Leukocytes, ×10^9/L	9 (7–12)	9 (7–12)	9 (7–12)	>0.9
Neutrophil count, ×10^9/L	6 (4–9)	6 (4–8)	7 (4–9)	0.6
Platelet count, ×10^9/L	301 (215–421)	332 (222–439)	241 (205–376)	0.055
Hb (mmol/L)	8 (7–9)	8 (7–9)	8 (7–9)	0.5
**Follow-up events**				
Leukopenia	27 (21%)	16 (19%)	11 (24%)	0.6
Neutropenia	63 (48%)	40 (47%)	23 (51%)	0.8
Thrombocytopenia	29 (22%)	17 (20%)	12 (27%)	0.5
Anemia	18 (14%)	9 (10%)	9 (20%)	0.2

Data presented as median (interquartile range) or *n* (%). ALT = alanine aminotransferase; AST = aspartate transaminase; eGFR = estimated glomerular filtration rate; gGT = gamma-glutamyl transferase; Hb = hemoglobulin.

Seventy-nine patients received a regimen consisting of 70 mg/m^2 cisplatin and 1000 mg/m^2 gemcitabine for an indication of bile duct or gallbladder cancer. The same dosages were used for an indication of bladder cancer (n = 40). A small group of patients (n = 10) were treated for an indication of non-small cell lung cancer with a regimen consisting of 75 mg/m^2 cisplatin and 1250 mg/m^2 gemcitabine. Details on remaining protocols are not provided due to a large diversification of protocols and patient pseudonymization.

Table S33 Baseline characteristics and follow-up events for patients with and without hepatic impairment receiving cyclophosphamide.

Baseline Characteristic	Overall n = 198	No Hepatic Impairment n = 160	Hepatic Impairment n = 38	p-Value
Age (years)	51 (38–60)	51 (40–61)	49 (35–59)	0.6
Male sex (%)	98 (49%)	85 (53%)	13 (34%)	0.036
ALT (U/L)	26 (17–38)	22 (16–31)	51 (34–102)	<0.001
AST (U/L)	22 (16–31)	19 (14–24)	52 (38–117)	<0.001
Bilirubin (µmol/L)	7 (6–9)	7 (6–9)	8 (6–11)	0.3
eGFR (ml/min)	90 (60–90)	90 (60–90)	90 (60–90)	>0.9
gGT (U/L)	32 (21–61)	31 (20–46)	42 (27–207)	0.006
Albumin (g/L)	35 (28–40)	37 (30–40)	31 (25–36)	0.002
Leukocytes, ×10^9/L	9 (6–13)	8 (6–12)	10 (7–14)	0.2
Neutrophil count, ×10^9/L	6 (4–10)	6 (4–9)	8 (5–11)	0.12
Platelet count, ×10^9/L	279 (224–354)	273 (224–333)	313 (227–420)	0.2
Hb (mmol/L)	8 (7–9)	8 (7–9)	7 (6–8)	0.002
**Follow-up events**				
Leukopenia	74 (37%)	68 (43%)	6 (16%)	0.004
Neutropenia	77 (39%)	70 (44%)	7 (18%)	0.007
Thrombocytopenia	71 (36%)	65 (41%)	6 (16%)	0.007
Anemia	35 (18%)	30 (19%)	5 (13%)	0.6

Data presented as median (interquartile range) or *n* (%). ALT = alanine aminotransferase; AST = aspartate transaminase; eGFR = estimated glomerular filtration rate; gGT = gamma-glutamyl transferase; Hb = hemoglobulin.

The largest group of patients (n = 75) received a regimen consisting of 750 mg/m^2 cyclophosphamide for indications of various cancer types. Sixty-six patients received 2000 mg/m^2 cyclophosphamide for stem cell mobilization in multiple myeloma. Twenty-nine patients received 500

mg/m² for an indication of proliferative lupus nephritis. Details on remaining protocols are not provided due to a large diversification of protocols and patient pseudonymization.

Table S34 Baseline characteristics and follow-up events for patients with and without hepatic impairment receiving cyclophosphamide + docetaxel + epirubicin + fluorouracil.

Baseline Characteristic	Overall *n* = 202	No Hepatic Impairment *n* = 177	Hepatic Impairment *n* = 25	*p*-Value
Age (years)	53 (47–59)	53 (47–60)	56 (50–58)	0.7
Male sex (%)	2 (1.0%)	2 (1.1%)	0 (0%)	>0.9
ALT (U/L)	20 (14–28)	17 (14–24)	37 (28–54)	<0.001
AST (U/L)	23 (19–27)	21 (18–25)	36 (32–42)	<0.001
Bilirubin (µmol/L)	8 (7–11)	8 (7–11)	7 (6–8)	0.036
eGFR (ml/min)	80 (60–90)	79 (60–90)	89 (60–90)	0.5
gGT (U/L)	23 (16–38)	21 (16–32)	38 (24–50)	0.001
Albumin (g/L)	42 (39–44)	42 (39–45)	43 (42–44)	0.5
Leukocytes, $\times 10^9$/L	7 (5–8)	7 (5–8)	6 (5–8)	0.4
Neutrophil count, $\times 10^9$/L	4 (3–5)	4 (3–5)	4 (3–5)	0.2
Platelet count, $\times 10^9$/L	245 (212–281)	242 (210–278)	255 (221–301)	0.10
Hb (mmol/L)	8 (8–9)	8 (8–9)	8 (8–9)	0.5
**Follow-up events**				
Leukopenia	90 (45%)	78 (44%)	12 (48%)	0.9
Neutropenia	122 (60%)	108 (61%)	14 (56%)	0.8
Thrombocytopenia	9 (4.5%)	8 (4.5%)	1 (4.0%)	>0.9
Anemia	5 (2.5%)	3 (1.7%)	2 (8.0%)	0.12

Data presented as median (interquartile range) or *n* (%). ALT = alanine aminotransferase; AST = aspartate transaminase; eGFR = estimated glomerular filtration rate; gGT = gamma-glutamyl transferase; Hb = hemoglobulin.

All patients received a regimen of 500 mg/m² cyclophosphamide, 100 mg/m² docetaxel, 100 mg/m² epirubicine, and 500 mg/m² fluorouracil for an indication of mamma carcinoma.

Table S35 Baseline characteristics and follow-up events for patients with and without hepatic impairment receiving cyclophosphamide + doxorubicin.

Baseline Characteristic	Overall *n* = 128	No Hepatic Impairment *n* = 101	Hepatic Impairment *n* = 27	*p*-Value
Age (years)	52 (47–59)	51 (46–58)	56 (50–64)	0.074
Male sex (%)	9 (7.0%)	7 (6.9%)	2 (7.4%)	>0.9
ALT (U/L)	21 (15–30)	18 (14–25)	32 (23–46)	<0.001
AST (U/L)	24 (18–31)	21 (16–25)	53 (36–67)	<0.001
Bilirubin (µmol/L)	7 (6–10)	7 (5–9)	9 (7–12)	0.008
eGFR (ml/min)	80 (60–90)	81 (60–90)	79 (60–90)	0.7
gGT (U/L)	30 (17–58)	24 (16–40)	88 (39–193)	<0.001
Albumin (g/L)	40 (36–43)	41 (38–44)	37 (34–40)	0.051
Leukocytes, $\times 10^9$/L	7 (6–9)	7 (6–9)	7 (6–10)	0.5
Neutrophil count, $\times 10^9$/L	5 (4–6)	5 (4–6)	5 (4–8)	0.4
Platelet count, $\times 10^9$/L	286 (221–340)	272 (223–333)	299 (195–354)	0.9
Hb (mmol/L)	8 (8–9)	8 (8–9)	8 (7–9)	0.088
**Follow-up events**				
Leukopenia	64 (50%)	48 (48%)	16 (59%)	0.4

Neutropenia	80 (63%)	64 (63%)	16 (59%)	0.9
Thrombocytopenia	23 (18%)	17 (17%)	6 (22%)	0.6
Anemia	18 (14%)	10 (9.9%)	8 (30%)	0.024

Data presented as median (interquartile range) or *n* (%). ALT = alanine aminotransferase; AST = aspartate transaminase; eGFR = estimated glomerular filtration rate; gGT = gamma-glutamyl transferase; Hb = hemoglobulin.

Almost all patients (117/128, 91%) receiving the combination therapy of cyclophosphamide + doxorubicin, were treated for an indication of mamma carcinoma. Dosing schedules included 600 mg/m^2 cyclophosphamide + 60 mg/m^2 doxorubicin administered 3 or 4 times a week for 2 to 3 weeks. Details on remaining protocols are not provided due to a large diversification of protocols and patient pseudonymization.

Table S36 Baseline characteristics and follow-up events for patients with and without hepatic impairment receiving cyclophosphamide + doxorubicin + rituximab + vincristine.

Baseline Characteristic	Overall *n* = 182	No Hepatic Impairment *n* = 133	Hepatic Impairment *n* = 49	*p*-Value
Age (years)	61 (49–70)	61 (47–70)	60 (54–69)	0.8
Male sex (%)	119 (65%)	88 (66%)	31 (63%)	0.7
ALT (U/L)	21 (15–37)	18 (14–25)	40 (22–60)	<0.001
AST (U/L)	24 (18–41)	21 (16–24)	50 (42–63)	<0.001
Bilirubin (µmol/L)	8 (6–11)	9 (7–11)	8 (5–12)	>0.9
eGFR (ml/min)	83 (60–90)	82 (60–90)	83 (60–90)	0.6
gGT (U/L)	33 (21–61)	28 (20–56)	53 (28–91)	<0.001
Albumin (g/L)	38 (34–42)	39 (35–42)	36 (33–41)	0.2
Leukocytes, ×10^9/L	9 (7–12)	9 (7–11)	9 (6–13)	0.8
Neutrophil count, ×10^9/L	6 (4–8)	5 (4–8)	6 (4–8)	>0.9
Platelet count, ×10^9/L	283 (221–375)	276 (217–384)	287 (227–348)	>0.9
Hb (mmol/L)	8 (7–9)	8 (7–9)	8 (7–8)	0.007
**Follow-up events**				
Leukopenia	99 (54%)	74 (56%)	25 (51%)	0.7
Neutropenia	128 (70%)	91 (68%)	37 (76%)	0.5
Thrombocytopenia	47 (26%)	31 (23%)	16 (33%)	0.3
Anemia	38 (21%)	25 (19%)	13 (27%)	0.4

Data presented as median (interquartile range) or *n* (%). ALT = alanine aminotransferase; AST = aspartate transaminase; eGFR = estimated glomerular filtration rate; gGT = gamma-glutamyl transferase; Hb = hemoglobulin.

All patients receiving this compound combination were treated with 750 mg/m^2 cyclophosphamide, 50 mg/m^2 doxorubicin, 375 mg/m^2 rituximab, and 2 mg/m^2 vincristine. This protocol was used for the indication of mantle cell lymphoma and diffuse large B-cell lymphoma.

Table S37 Baseline characteristics and follow-up events for patients with and without hepatic impairment receiving cyclophosphamide + fludarabine.

Baseline Characteristic	Overall *n* = 47	No Hepatic Impairment *n* = 35	Hepatic Impairment *n* = 12	*p*-Value
Age (years)	53 (39–62)	57 (41–62)	42 (22–54)	0.039
Male sex (%)	25 (53%)	19 (54%)	6 (50%)	0.8
ALT (U/L)	26 (16–50)	22 (14–29)	58 (47–77)	<0.001
AST (U/L)	26 (22–31)	23 (20–26)	45 (33–92)	<0.001

Bilirubin (μmol/L)	9 (6–14)	9 (6–12)	13 (8–26)	0.061
eGFR (ml/min)	89 (60–90)	83 (60–90)	90 (60–90)	0.5
gGT (U/L)	36 (27–71)	32 (24–53)	86 (35–139)	0.004
Albumin (g/L)	41 (39–43)	40 (39–42)	41 (36–44)	0.9
Leukocytes, $\times 10^9$/L	6 (5–7)	6 (4–7)	6 (5–7)	0.7
Neutrophil count, $\times 10^9$/L	4 (2–5)	4 (2–5)	4 (3–5)	0.6
Platelet count, $\times 10^9$/L	213 (139–280)	216 (164–281)	174 (86–273)	0.3
Hb (mmol/L)	7 (6–8)	7 (7–8)	7 (6–8)	0.7
**Follow-up events**				
Leukopenia	46 (98%)	35 (100%)	11 (92%)	0.3
Neutropenia	46 (98%)	35 (100%)	11 (92%)	0.3
Thrombocytopenia	35 (74%)	25 (71%)	10 (83%)	0.7
Anemia	26 (55%)	17 (49%)	9 (75%)	0.2

Data presented as median (interquartile range) or *n* (%). ALT = alanine aminotransferase; AST = aspartate transaminase; eGFR = estimated glomerular filtration rate; gGT = gamma-glutamyl transferase; Hb = hemoglobulin.

Out of 47 patients, 25 patients received a regimen consisting of 60 mg/m^2 cyclophosphamide and 40 mg/m^2 fludarabine for allogeneic stem cell transplantation. Sixteen patients received a regimen of 500 mg/m^2 cyclophosphamide and 30 mg/m^2 fludarabine for an indication of non-Hodgkin lymphoma. Details on remaining protocols are not provided due to a large diversification of protocols and patient pseudonymization.

Table S38 Baseline characteristics and follow-up events for patients with and without hepatic impairment receiving doxorubicin.

Baseline Characteristic	Overall n = 38	No Hepatic Impairment n = 26	Hepatic Impairment n = 12	p-Value
Age (years)	62 (54–68)	62 (54–67)	61 (56–71)	0.6
Male sex (%)	9 (24%)	8 (31%)	1 (8.3%)	0.2
ALT (U/L)	20 (13–29)	18 (13–24)	30 (19–57)	0.031
AST (U/L)	23 (18–39)	20 (18–22)	48 (37–64)	<0.001
Bilirubin (μmol/L)	8 (6–9)	7 (6–8)	9 (6–13)	0.3
eGFR (ml/min)	83 (69–90)	84 (73–90)	82 (64–90)	0.6
gGT (U/L)	50 (24–102)	36 (20–59)	112 (28–163)	0.010
Albumin (g/L)	39 (36–42)	41 (38–42)	37 (34–39)	0.2
Leukocytes, $\times 10^9$/L	9 (6–11)	9 (6–11)	8 (7–11)	>0.9
Neutrophil count, $\times 10^9$/L	5 (4–7)	6 (4–8)	5 (4–7)	0.6
Platelet count, $\times 10^9$/L	296 (244–401)	289 (247–401)	308 (196–403)	0.8
Hb (mmol/L)	8 (7–9)	8 (8–9)	7 (6–8)	0.001
**Follow-up events**				
Leukopenia	12 (32%)	7 (27%)	5 (42%)	0.5
Neutropenia	16 (42%)	9 (35%)	7 (58%)	0.3
Thrombocytopenia	4 (11%)	0 (0%)	4 (33%)	0.007
Anemia	7 (18%)	1 (3.8%)	6 (50%)	0.002

Data presented as median (interquartile range) or *n* (%). ALT = alanine aminotransferase; AST = aspartate transaminase; eGFR = estimated glomerular filtration rate; gGT = gamma-glutamyl transferase; Hb = hemoglobulin.

The majority of patients (n = 33) received a regimen consisting of 75 mg/m^2 for an indication of sarcoma. Remaining patients received 20 mg/m^2 for the treatment of mamma carcinoma.

Table S39 Baseline characteristics and follow-up events for patients with and without hepatic impairment receiving fluorouracil + irinotecan + oxaliplatin.

Baseline Characteristic	Overall $n = 53$	No Hepatic Impairment $n = 34$	Hepatic Impairment $n = 19$	p-Value
Age (years)	57 (51–64)	57 (50–64)	57 (53–66)	0.6
Male sex (%)	36 (68%)	24 (71%)	12 (63%)	0.6
ALT (U/L)	39 (25–62)	30 (20–48)	60 (39–143)	0.001
AST (U/L)	32 (24–42)	26 (22–32)	50 (40–67)	<0.001
Bilirubin (μmol/L)	9 (7–12)	8 (7–10)	12 (8–38)	<0.001
eGFR (ml/min)	90 (78–90)	90 (60–90)	90 (90–90)	0.2
gGT (U/L)	87 (42–259)	61 (28–161)	252 (55–508)	0.004
Albumin (g/L)	40 (36–43)	40 (35–43)	39 (37–43)	0.7
Leukocytes, $\times 10^9$/L	8 (7–10)	8 (7–10)	8 (6–9)	0.3
Neutrophil count, $\times 10^9$/L	6 (4–7)	6 (4–8)	5 (4–6)	0.3
Platelet count, $\times 10^9$/L	269 (205–297)	256 (205–292)	270 (184–355)	0.7
Hb (mmol/L)	8 (8–9)	8 (8–9)	8 (8–9)	0.7
**Follow-up events**				
Leukopenia	12 (23%)	9 (26%)	3 (16%)	0.5
Neutropenia	20 (38%)	11 (32%)	9 (47%)	0.4
Thrombocytopenia	3 (5.7%)	2 (5.9%)	1 (5.3%)	>0.9
Anemia	3 (5.7%)	2 (5.9%)	1 (5.3%)	>0.9

Data presented as median (interquartile range) or *n*(%). ALT = alanine aminotransferase; AST = aspartate transaminase; eGFR = estimated glomerular filtration rate; gGT = gamma-glutamyl transferase; Hb = hemoglobulin.

Patients were treated with a standard FOLFIRINOX protocol consisting of 400 + 2400 mg/m^2 fluorouracil, 180 mg/m^2 irinotecan, and 85 mg/m^2 oxaliplatin for an indication of pancreatic cancer.

Table S40 Baseline characteristics and follow-up events for patients with and without hepatic impairment receiving gemcitabine.

Baseline Characteristic	Overall $n = 58$	No Hepatic Impairment $n = 35$	Hepatic Impairment $n = 23$	p-Value
Age (years)	66 (59–72)	67 (61–73)	62 (56–67)	0.058
Male sex (%)	33 (57%)	23 (66%)	10 (43%)	0.094
ALT (U/L)	28 (20–51)	25 (16–29)	69 (27–116)	<0.001
AST (U/L)	30 (21–46)	22 (18–29)	55 (40–73)	<0.001
Bilirubin (μmol/L)	8 (6–13)	8 (6–11)	8 (6–17)	0.5
eGFR (ml/min)	85 (60–90)	88 (60–90)	69 (60–90)	0.5
gGT (U/L)	68 (43–135)	60 (38–124)	100 (59–335)	0.010
Albumin (g/L)	36 (33–40)	37 (34–40)	36 (31–38)	0.3
Leukocytes, $\times 10^9$/L	8 (7–10)	8 (7–11)	7 (6–9)	0.3
Neutrophil count, $\times 10^9$/L	4 (4–6)	5 (4–7)	4 (3–6)	0.2
Platelet count, $\times 10^9$/L	305 (247–398)	292 (249–398)	310 (233–416)	>0.9
Hb (mmol/L)	8 (7–8)	7 (7–8)	8 (7–8)	0.8
**Follow-up events**				
Leukopenia	9 (16%)	4 (11%)	5 (22%)	0.5
Neutropenia	37 (64%)	19 (54%)	18 (78%)	0.11
Thrombocytopenia	6 (10%)	4 (11%)	2 (8.7%)	>0.9
Anemia	6 (10%)	4 (11%)	2 (8.7%)	>0.9

Data presented as median (interquartile range) or *n* (%). ALT = alanine aminotransferase; AST = aspartate transaminase; eGFR = estimated glomerular filtration rate; gGT = gamma-glutamyl transferase; Hb = hemoglobulin.

Patients received a regimen of 1000 mg/m² gemcitabine for an indication of pancreatic cancer.

Table S41 Baseline characteristics and follow-up events for patients with and without hepatic impairment receiving melphalan.

Baseline Characteristic	Overall *n* = 152	No Hepatic Impairment *n* = 139	Hepatic Impairment *n* = 13	*p*-Value
Age (years)	60 (56–64)	60 (56–64)	59 (53–60)	0.12
Male sex (%)	107 (70%)	98 (71%)	9 (69%)	>0.9
ALT (U/L)	18 (13–24)	17 (13–23)	31 (20–55)	<0.001
AST (U/L)	19 (14–24)	19 (14–23)	40 (27–62)	<0.001
Bilirubin (µmol/L)	7 (6–10)	7 (5–9)	9 (6–29)	0.067
eGFR (ml/min)	88 (60–90)	90 (60–90)	76 (60–90)	0.6
gGT (U/L)	32 (21–47)	31 (21–43)	57 (35–110)	0.015
Albumin (g/L)	40 (37–42)	40 (37–42)	39 (36–43)	0.6
Leukocytes, ×10⁹/L	6 (5–7)	6 (5–7)	4 (4–7)	0.10
Neutrophil count, ×10⁹/L	4 (3–5)	4 (3–5)	2 (2–4)	0.020
Platelet count, ×10⁹/L	249 (205–321)	254 (207–321)	243 (169–340)	0.4
Hb (mmol/L)	8 (7–9)	8 (7–9)	9 (8–9)	0.023
**Follow-up events**				
Leukopenia	51 (34%)	42 (30%)	9 (69%)	0.010
Neutropenia	53 (35%)	44 (32%)	9 (69%)	0.012
Thrombocytopenia	51 (34%)	42 (30%)	9 (69%)	0.010
Anemia	14 (9.2%)	12 (8.6%)	2 (15%)	0.3

Data presented as median (interquartile range) or *n* (%). ALT = alanine aminotransferase; AST = aspartate transaminase; eGFR = estimated glomerular filtration rate; gGT = gamma-glutamyl transferase; Hb = hemoglobulin.

Out of 152 patients, 141 patients were treated with 100 mg/m² for an indication of autologous stem cell transplantation in multiple myeloma patients. Remaining patients were treated with 70 mg/m² for various indications.

Table S42 Baseline characteristics and follow-up events for patients with and without hepatic impairment receiving methotrexate.

Baseline Characteristic	Overall *n* = 76	No Hepatic Impairment *n* = 59	Hepatic Impairment *n* = 17	*p*-Value
Age (years)	61 (37–68)	61 (34–68)	59 (47–68)	0.7
Male sex (%)	42 (55%)	32 (54%)	10 (59%)	0.7
ALT (U/L)	25 (16–44)	22 (15–30)	53 (42–84)	<0.001
AST (U/L)	22 (17–31)	19 (16–24)	60 (34–80)	<0.001
Bilirubin (µmol/L)	9 (8–13)	8 (7–12)	11 (9–13)	0.030
eGFR (ml/min)	90 (66–90)	90 (75–90)	90 (60–90)	0.6
gGT (U/L)	35 (21–65)	29 (18–40)	100 (51–171)	<0.001
Albumin (g/L)	36 (34–39)	37 (35–40)	34 (30–38)	0.061
Leukocytes, ×10⁹/L	7 (5–10)	7 (5–11)	7 (4–8)	0.3
Neutrophil count, ×10⁹/L	5 (3–8)	5 (3–9)	5 (3–7)	0.6

Platelet count, ×10⁹/L	245 (187–310)	254 (188–309)	222 (121–311)	0.3
Hb (mmol/L)	8 (7–9)	8 (7–9)	7 (6–8)	0.001
**Follow-up events**				
Leukopenia	43 (57%)	29 (49%)	14 (82%)	0.031
Neutropenia	44 (58%)	30 (51%)	14 (82%)	0.041
Thrombocytopenia	35 (46%)	24 (41%)	11 (65%)	0.14
Anemia	30 (39%)	22 (37%)	8 (47%)	0.7

Data presented as median (interquartile range) or *n* (%). ALT = alanine aminotransferase; AST = aspartate transaminase; eGFR = estimated glomerular filtration rate; gGT = gamma-glutamyl transferase; Hb = hemoglobulin.

Out of 76 patients, only 20 patients were treated with a high dose of 3000 mg/m² methotrexate. Remaining patients were treated with low-dose methotrexate (1–15 mg/m²) for various indications.

Table S43 Baseline characteristics and follow-up events for patients with and without hepatic impairment receiving paclitaxel.

Baseline Characteristic	Overall *n* = 61	No Hepatic Impairment *n* = 34	Hepatic Impairment *n* = 27	*p*-Value
Age (years)	57 (49–67)	63 (49–71)	55 (48–64)	0.036
Male sex (%)	6 (9.8%)	6 (18%)	0 (0%)	0.030
ALT (U/L)	23 (15–32)	18 (13–25)	29 (19–41)	0.001
AST (U/L)	28 (21–45)	21 (18–26)	45 (36–55)	<0.001
Bilirubin (μmol/L)	8 (6–11)	8 (6–10)	9 (7–13)	0.10
eGFR (ml/min)	87 (60–90)	86 (58–90)	90 (60–90)	0.3
gGT (U/L)	39 (28–92)	37 (21–47)	80 (30–147)	0.017
Albumin (g/L)	39 (37–41)	39 (38–42)	38 (35–40)	0.083
Leukocytes, ×10⁹/L	7 (6–9)	7 (6–9)	7 (6–10)	>0.9
Neutrophil count, ×10⁹/L	5 (3–6)	4 (3–6)	5 (3–7)	0.4
Platelet count, ×10⁹/L	286 (240–340)	284 (244–340)	292 (196–345)	0.6
Hb (mmol/L)	8 (7–8)	8 (7–8)	7 (7–8)	0.083
**Follow-up events**				
Leukopenia	11 (18%)	4 (12%)	7 (26%)	0.2
Neutropenia	16 (26%)	8 (24%)	8 (30%)	0.8
Thrombocytopenia	2 (3.3%)	1 (2.9%)	1 (3.7%)	>0.9
Anemia	6 (9.8%)	3 (8.8%)	3 (11%)	>0.9

Data presented as median (interquartile range) or *n* (%). ALT = alanine aminotransferase; AST = aspartate transaminase; eGFR = estimated glomerular filtration rate; gGT = gamma-glutamyl transferase; Hb = hemoglobulin.

The majority of patients (*n* = 56) received a regimen consisting of 80 or 90 mg/m² paclitaxel for an indication of mamma carcinoma. Details on remaining protocols are not provided due to a large diversification of protocols and patient pseudonymization.
